# Plasma lipidomics and 15-year risk of incident diabetes: a coronary artery risk development in young adults study

**DOI:** 10.1016/j.jlr.2026.100992

**Published:** 2026-01-30

**Authors:** Jessica K. Sprinkles, Annie Green Howard, Autumn G. Hullings, Aditya Shetye, John T. Wilkins, Misa Graff, Saame Raza Shaikh, Christy L. Avery, Kari E. North, Penny Gordon-Larsen, Katie A. Meyer

**Affiliations:** 1 Department of Nutrition, Gillings School of Global Public Health, University of North Carolina at Chapel Hill, Chapel Hill, NC, USA; 2 Department of Biostatistics, Gillings School of Global Public Health, University of North Carolina at Chapel Hill, Chapel Hill, NC, USA; 3 Carolina Population Center, University of North Carolina at Chapel Hill, Chapel Hill, NC, USA; 4 Division of Cardiology, Feinberg School of Medicine, Northwestern University, Chicago, IL, USA; 5 Department of Preventive Medicine, Feinberg School of Medicine, Northwestern University, Chicago, IL, USA; 6 Department of Epidemiology, Gillings School of Global Public Health, University of North Carolina at Chapel Hill, Chapel Hill, NC, USA; 7 Department of Epidemiology, The University of Texas Health Science Center at Houston School of Public Health, Brownsville Regional Campus, Brownsville, TX, USA; 8 Nutrition Research Institute, University of North Carolina at Chapel Hill, Kannapolis, NC, USA

**Keywords:** lipidome, epidemiology, nutrition, metabolism, ceramides, triacylglycerol, diabetes, cohort study

## Abstract

Lipid metabolism has long been implicated in diabetes, but there has been a paucity of population-based studies of the plasma lipidome and incident diabetes in cohorts of early middle age. We used data from the US-based Coronary Artery Risk Development in Young Adults (CARDIA) Study to identify lipidomics associated with 15-year incident diabetes (n = 1,094; n = 162 incident diabetes; [mean (SD) age: 45 (3.6); 58% women; and 59% White race]). Plasma lipidomics was conducted using liquid-chromatography and infusion-mass spectrometry. Diabetes was defined at 5-, 10- and 15-year follow-ups as fasting glucose ≥ 126 mg/dl, 2-h glucose tolerance test ≥ 200 mg/dl, HbA1c ≥ 6.5%, or reported diabetic medication use. We tested associations between individual lipids and incident diabetes with interval-censored, multivariable-adjusted Cox proportional hazards regression, accounting for multiple comparisons. We used differential expression analysis to identify pathways upregulated and downregulated in participants who developed diabetes over the 15-year period. Finally, we used penalized regression (LASSO) to generate a lipid risk score for incident diabetes (0.7 training, 0.3 testing). In hazards regression, 156 lipids including glycerolipids, glycerophospholipids, and sphingolipids, were associated with incident diabetes. Of these, 56 lipids were also selected by LASSO regression as distinguishing participants who developed diabetes from those who did not. The lipid risk score’s ability to improve prediction of 15-year incident diabetes past sociodemographic, behavioral, and clinical covariates was limited to the training set. Pathways leading to diacylglycerols and ceramides were upregulated, while pathways leading to hexosylceramides, lysophosphatidylethanolamines, triacylglycerols, and lysophosphatidylcholines were downregulated in incident diabetes cases. Our results in this cohort of early middle-aged adults, supports further investigation into the roles of glycerophospholipid and sphingolipid metabolism in diabetes development, particularly for ceramides and hexosylceramides.

## Graphical Abstract

**Figure:**
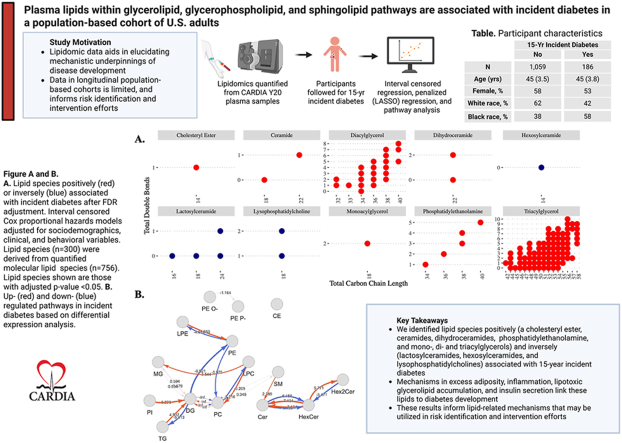

The prevalence of diabetes in the United States has continued to rise (1). Lipid-related metabolic dysregulation in diabetes increases the risk of complications, including cardiovascular disease—the leading cause of death in the United States (1, 2). Early identification and intervention for diabetes is critical. Diabetes risk is currently assessed through standard clinical measures, including HDL-c and total triacylglycerols (TAGs), though these measures may lack prognostic specificity (3). In contrast to standard clinical measures, lipid species may serve as mechanistically relevant biomarkers of T2D. For example, ceramide (CER) species directly influence insulin signaling (4), lysophosphatidylcholine (LPC) species stimulate glucose uptake (5), and diacylglycerol (DAG) species induce insulin-resistance (6), contributing to T2D. Lipidomic data that capture systemic lipid abnormalities, which often precede observable phenotypic changes, shows promise for improving risk prediction (2).

Plasma lipidomic studies, or the study of the molecular lipid species present in human plasma, offer a more granular approach to assessing diabetes risk, compared to standard clinical measures. Lipid species can reflect underlying diabetes-related mechanisms, including aspects of glucose-metabolism and insulin resistance, allowing these data to distinguish prediabetes from diabetes (7) and predict cardiovascular disease in individuals with diabetes (8). Although these inform the use of lipidomic data in prevalent diabetes, the ability of these data to determine diabetes risk in healthy individuals is not well understood. Population-based evidence of the lipidome prior to onset of diabetes at early middle age is needed to advance the clinical utility of these data (9, 10). Current lipidomic studies of incident diabetes have been largely limited to nested case-control studies (11, 12, 13, 14, 15, 16), samples of older baseline age (8, 15), or test a relatively small number of lipids (17, 18, 19).

In the present study, we examined the plasma lipidome prior to diabetes onset in a population-based cohort study of early middle-aged US adults. We quantified 756 molecular lipid species across 14 lipid classes at baseline and tested associations with 15-year incident diabetes. We further identified lipid pathways that are dysregulated prior to onset of diabetes. Finally, we derived a lipidomic risk score (LRS) to evaluate the ability of multivariate statistical approaches to distinguish participants who do and do not develop incident diabetes. We hypothesized that lipid species within the classes of TAGs, DAGs, phosphatidylcholines (PC), phosphatidylethanolamines (PEs), sphingomyelins (SMs), and CER are positively associated with incident diabetes; and that species within the classes of LPCs and lactosylceramides (LCERs) are inversely associated with incident diabetes. Furthermore, we expected that the addition of a LRS, derived from supervised analysis, improves prediction of incident diabetes beyond standard sociodemographic, health, and clinical variables. Our results inform the identification of lipidomic biomarkers that precede diabetes onset and lipid-related pathways underpinning diabetes development in a US population-based study of early middle aged adults, which is crucial, as the age of diabetes onset is decreasing.

## Materials and Methods

### Study sample

Beginning in 1985–86, the Coronary Artery Risk Development in Young Adults (CARDIA) study is a population-based, longitudinal cohort study that recruited males and females of self-reported Black and White race from four US urban centers (Birmingham, AL; Chicago, IL; Minneapolis, MN; Oakland, CA). Across 9 follow-up examinations, clinic staff have collected data related to the evolution of cardiometabolic diseases and their risk factors. Examination protocols were approved by the Institutional Review Board at each CARDIA site; each participant provided active, signed consent, and the study was conducted in accordance with the Declaration of Helsinki.

We used plasma lipidomic from the year 20 exam (2005–2006), and diabetes data from years 20, 25, 30, and 35 of follow-up. Lipidomics data were generated for 1,341 participants. We excluded participants with diabetes at year 20 (n = 96), leaving an analytic sample of 1,245 (186 incident diabetes cases) participants. Other exclusions were due to missing covariate data, and varied across analysis. In multivariable-adjusted models, the analytic sample was limited to those with complete covariate data (n = 1,094; incident diabetes cases = 162), excluding 151 participants (n missing: current smoking = 15, physical activity = 4, dietary intake = 130, hypertension = 10, and lipid-lowering medication use = 4) (Supplemental Fig. S1).

### Blood collection and lipidomic measures

Prior to examination, participants were asked to avoid physical activity and smoking for ≥2 h, and fast for ≥12 h. Blood was drawn by venipuncture, separated through centrifugation, aliquoted, flash-frozen, and stored at −70°C. Lipidomic data were generated by Metabolon, using their Targeted Complex Lipid Panel. Lipids were extracted and concentrated using internal standards. A Shimadzu LC with nano PEEK tubing and the Sciex SelexION-5500 QTRAP were used for infusion-MS in both positive and negative mode electrospray. Intensity ratios of target compounds and their assigned internal standards were used to quantify molecular lipid species. Lipidomics yielded quantification of 756 molecular lipid species across 14 lipid classes: TAGs, DAGs, monoacylglycerols (MAGs), CERs, hexosylceramides (HCER), LCERs, dihydroceramides (DCERs), PCs, PEs, phosphatidylinositols (PIs), LPCs, lysophosphatidylethanolamines (LPEs), SMs, and cholesterol esters (CEs).

### Diabetes assessment

Diabetes status was assessed at each CARDIA examination as fasting glucose ≥ 126 mg/dl, 2-h oral glucose tolerance test ≥ 200 mg/dl, hemoglobin HbA1c ≥ 6.5%, or reported use of diabetes medication.

### Covariate measures

Interviewer-administered questionnaires were used to assess sociodemographic characteristics (age, race, sex, and education), smoking status, and medication use (including lipid-lowering, anti-hypertensive, and diabetes-related). A validated interviewer-administered CARDIA Physical Activity History questionnaire collected data on engagement in 13 activities over the past year, from which the CARDIA total physical activity score was calculated. The total physical activity score (Exercise Units) considers both reported frequency (hours/week) and intensity (METs/minute) of activities. Dietary intake was assessed with an interviewer-administered diet history questionnaire, capturing past 28-day intake, at year 20. Food group data, assessed as servings per day, were generated by the University of Minnesota Nutrition Coordinating Center. We used the following food groups, in addition to total calories (kcal/day) and the CARDIA a priori diet quality score (APDQS), in our analysis: eggs, processed meat, lean red meat, regular red meat, poultry, refined grains, whole grains, seeds and nuts, green vegetables, yellow vegetables, other vegetables, fruit, fried foods, and fatty fish.

Clinical measures were assessed with standardized and validated protocols by trained study staff. Weight and height were measured in the clinic using a calibrated scale. Blood pressure was measured with a random zero sphygmomanometer, using the average of the final two of three measures. Hypertension was defined as: a systolic pressure ≥ 140 mmHg, a diastolic pressure of ≥ 90 mmHg, or reported use of anti-hypertensive medication. The estimated glomerular filtration rate (eGFR, mm/min/1.32 m^2^) was calculated from age, race, sex, and serum creatinine using the 2009 Chronic Kidney Disease Epidemiology Collaboration equation.

### Statistical analysis

Molecular lipid species with missing data in >25% of the plasma samples were excluded and those with missing data in <25% of plasma samples were imputed with values generated from a random uniform distribution between the lowest and one-tenth of the lowest observed concentration. In addition, and in accordance with previous studies (13), 300 lipid species were derived from 756 molecular lipid species to reflect the total concentration of lipids with a common head group, total number of carbons, and total number of double bonds. This derivation applies only to lipids containing >1 FA. For lipids containing 1 FA, lipid species are synonymous with molecular lipid species. For example, lipid species PC (38:4) is the sum of molecular lipid species PC (16:0/22:4), PC (18:0/20:4), and PC (18:1/20:3). These 300 lipid species were used in individual regressions and in supplemental LRS derivation to increase comparability to literature. All lipid variables were log2 transformed and z-scaled.

Statistical analysis adjusted for sociodemographics [field center (Birmingham, AL; Chicago, IL; Minneapolis, MN; Oakland, CA), age (continuous), sex (male/female), self-reported race (Black/White), and highest education attained (continuous years)], health behaviors [physical activity score (continuous), smoking (current yes/no), and diet (food groups, APDQS, and total energy)], and clinical measures [eGFR (continuous), BMI (continuous), hypertension (yes/no), and lipid-lowering medication use (yes/no)].

Interval censored Weibull-Cox proportional hazards regression models estimated hazard ratios (HRs) (95% CI) for associations between each of the 300 lipid species and 15-year incident diabetes. In models of TAG species, an additional model included adjustment for total TAGs. This allowed testing TAG species composition, which has revealed distinct associations in some populations (20, 21, 22). Analysis was also conducted on lipid classes. *P* values and 95% CIs were adjusted for multiple comparisons using the false discovery rate (FDR) at significance threshold q < 0.05.

Differential expression analysis was conducted to identify lipid species that exhibited significant differences between participants who did and did not develop diabetes over the 15-year follow-up period. Significant species from unadjusted differential expression analysis (q-values < 0.05) were used to identify lipid pathways that distinguished participants who did and did not develop diabetes. Pathway activity and lipid reaction networks were generated for visualization. These analyses were conducted using the “LipidSigR” package in R. (23).

Two complementary multivariate statistical analyses were used to examine the lipidome with respect to incident diabetes. Principal components analysis (PCA), a data reduction approach, was used to generate orthogonal PCs that explained variability in the 756 molecular lipid species. PCs were evaluated using the scree plot, variability explained, and substantive considerations. Selected PCs were entered into multivariable-adjusted hazard regression models to test their association with incident diabetes. This unsupervised analysis reflects the correlational structure and variability in the lipids without consideration of the outcome. Next, penalized regression with a least absolute shrinkage and selection operator (LASSO) was used to select lipid species that distinguished participants who developed diabetes and to derive a LRS from selected variables. This supervised analysis served as a data reduction method for selection of uncorrelated lipids that explain variability in the outcome. The LASSO regression model was trained in a case-balanced 0.7 subset to balance those with (cases) and without (noncases) diabetes. The lambda parameter was determined through 10-fold cross validation with area under the curve (AUC) as the loss function. Using the minimum lambda, we tested this model on the remaining 0.3 (case-unbalanced) subset. The case-balanced 0.7 split and the 0.3 split had 423/448 and 56/318 with/without diabetes, respectively. Beta coefficients from the LASSO selected lipid species were used to derive a weighted LRS, calculated as the sum of the product of each lipid concentration and that lipids beta coefficient, divided by the total number of selected lipids. To evaluate how the LRS and sets of covariates impacted prediction of diabetes, the AUC was compared for the following models, with respect to 15-year risk of incident diabetes: Model 1: LRS; Model 2: health behaviors and clinical measures; Model 3: sociodemographics; Model 4: sociodemographics + health and clinical measures; Model 5: sociodemographics + health and clinical measures + LRS. We restricted covariates in this analysis to those reasonably available in a clinical setting. Each set of models was tested in both the 0.7 training data and the 0.3 testing data. Our main analysis focused on the addition of a lipid species-based LRS; in sensitivity analysis, we tested the addition of a molecular lipid species-based LRS. Additional methodologic details related to the CARDIA study, covariate measures, Metabolon, LIPID MAPS, and statistical methods are available in the technical supplement.

CARDIA data are available upon request from the CARDIA Coordinating Center, using guidance provided at: http://www.cardia.dopm.uab.edu/publications-2/publications-documents. CARDIA data are also publicly available on the NIH-supported BioLINCC and dbGaP platforms.

## Results

### Participant characteristics

Over the 15-year study period, 186 of the 1,245 participants developed diabetes. As compared to participants who did not develop diabetes, incident diabetes was positively associated with self-reported Black race, current smoking, BMI, hypertension, and lipid-lowering medication use; and inversely associated with physical activity, the APDQS, and eGFR (Table 1). Participants who developed incident diabetes had higher baseline (year 20) concentrations of lipid classes DAG, DCER, and TAG; and lower concentrations of HCER, LCER, LPC, and LPE; as compared to those who did not develop diabetes (Supplemental Table S1).

**Table 1:** Participant characteristics^a^ according to 15-year incident diabetes: CARDIA 2005–2021

Characteristic	15-yr incident diabetes		*P* value^b^
	No	Yes	
n	1,059	186	
Female, %	58	53	0.26
White race, %	62	42	<0.001
Age (years)	45 (3.5)	45 (3.8)	0.58
Education attained (years)	16.19 (2.53)	15.37 (2.46)	<0.001
Current smoking, %	15	26	<0.001
Total physical activity score, median (IQR)	296 (135, 522)	243 (126, 452)	0.028
APDQS	62.97 (12.85)	56.82 (11.93)	<0.001
BMI (kg/m^2^)	27.75 (5.76)	32.83 (6.93)	<0.001
Hypertension, %	15	34	<0.001
Lipid-lowering medication use, %	6.1	14	<0.001
eGFR (mL/min/1.73 m^2^)	95.43 (20.83)	99.43 (20.69)	0.014

APDQS, a priori diet quality score; CARDIA, Coronary Artery Risk Development in Young Adults; IQR, interquartile range.

a Mean (SD), unless otherwise noted.

b Kruskal–Wallis rank sum test and Pearson’s Chi-square test for continuous and categorical variables, respectively.

### Lipid classes and species associations

In interval-censored Weibull-Cox proportional hazards models adjusted for sociodemographics, health behaviors, and clinical variables, TAG and DAG classes were positively associated with incident diabetes after FDR adjustment (Supplemental Table S2). Other classes were not significantly associated with incident diabetes.

**Fig. 1:**
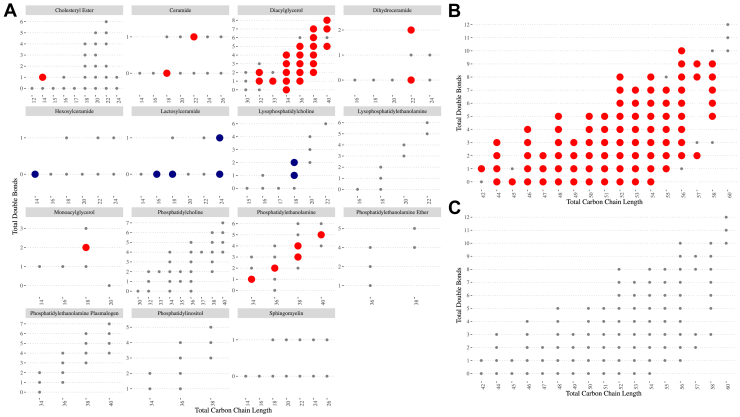
A-C: Multivariable-adjusted hazard ratios between lipid species and 15-year incident diabetes for (A) all lipid species, (B) TAG species, and (C) TAG species adjusted for total TAGs. Interval censored (Weibull) Cox hazards regression models adjusted for sociodemographics (sex, race, age, and education), health behaviors (current smoking, physical activity, and dietary intake), and clinical variables (BMI, hypertension, and lipid-lowering medication use). Significant associations (FDR adjusted q < 0.05) are indicated by dot size and color: large red dots = positively associated, large navy dots = inversely associated, small gray dots: not associated. n total/cases = 1,094/162. TAG, triacylglycerol; FDR, false discovery rate.

**Fig. 2:**
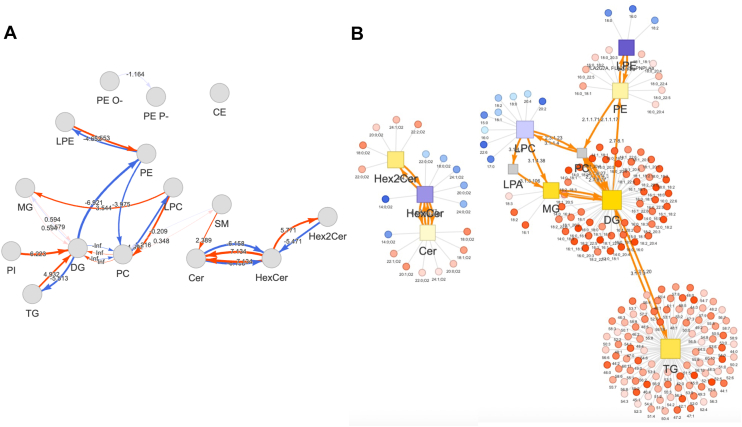
A-B: Differentially expressed lipid species in participants who did or did not develop diabetes over the 15-year study period. A: Pathway activity network identified upregulated or downregulated pathways between lipid classes. Red and blue arrows indicate upregulated and downregulated pathways, respectively. Pathway activity scores quantify magnitude of activity changes across pathways (0 = no change). B: Lipid reaction network displays significant lipid classes (square nodes) and lipid species (circular nodes). Upregulated classes and species are indicated by yellow squares and red circles, respectively. Downregulated class and species are indicated by purple squares and blue circles, respectively. Gray squares are insignificant or missing data. Strength of associations are indicated by color darkness (darker = more significant).

Of the 300 lipid species, 156 were associated with incident diabetes, after FDR adjustment. Lipid species within classes CE, CER, DCER, PE, DAG, MAG, and TAG were positively, and those within classes HCER, LCER, and LPC were inversely, associated with incident diabetes (Fig. 1A; Supplemental Table S3). Most TAG species were positively associated with incident diabetes (86 of 96); TAGs that were not associated with diabetes tended to contain longer, more polyunsaturated FAs (total double bonds > 7 or total carbon length > 55) (Fig. 1B). After adjustment for total TAGs, no TAG species remained statistically significant (Fig. 2C).

### Lipid pathway analysis

In differential expression analysis, 234 lipid species significantly differed among those who developed diabetes, compared to those who did not, over the 15-year follow-up period. Of the 234 lipid species, most were TAGs and DAGs, followed by PCs, and CEs (Supplemental Table S4). Based on the magnitude of the fold change, the top ten lipids upregulated in incident diabetes included DAG (16:1/16:1), DAG (16:0/16:1), DAG (16:1/18:0), cholesteryl ester [CE] (22:2), TAG (45:0), DAG (16:0/18:3), DAG (16:0/16:0), TAG (52:8), DAG (16:0/20:4), DAG (14:0/18:1), and the top ten lipids downregulated in incident diabetes included HCER (14:0), LCER (14:0), LPC (22:6), LCER (24:1), LPC (20:2), LCER (24:0), PC (18:2/18:3), LPE (16:0), LPC (17:0), and LPC (15:0) (Supplemental Fig. S2B).

Lipid pathways leading to DAG and CER production were upregulated, while pathways leading to HCER, LPE, TAG, and LPC production were downregulated, among those with incident diabetes (Fig. 2A). The pathway leading from PEs to PCs was downregulated, but the pathway leading from LPCs to PCs was upregulated, in diabetes; similarly, the pathway leading from DAGs to PEs was downregulated, but the pathway leading from LPEs to PEs was upregulated, in diabetes. Significant lipid species within these pathways are shown in Fig. 2B. Most notably, and consistent with upregulated pathways leading to DAG synthesis, many DAG species were highly significant in differential expression analysis.

### PCA of molecular lipid species

In PCA of the 756 molecular lipid species, the first PC explained 46.2% and the second PC explained 6.5% of variability (Supplemental Fig. S3). In multivariable adjusted interval censored Cox models, the PC1 and PC2 were associated with incident diabetes [HR (95% CI): PC1 = 0.98 (0.97, 0.99), PC2 = 1.03 (1.01, 1.05)]. Differences in PC1 and PC2 are due to TAG FA composition. Both PCs are inversely loaded with short, saturated TAGs; No TAGs load positively on PC1, while long, PUFA-containing TAGs and DAGs load positively on PC2 (Supplemental Fig. S4A, B).

### Penalized (LASSO) regression derived LRS

**Fig. 3:**
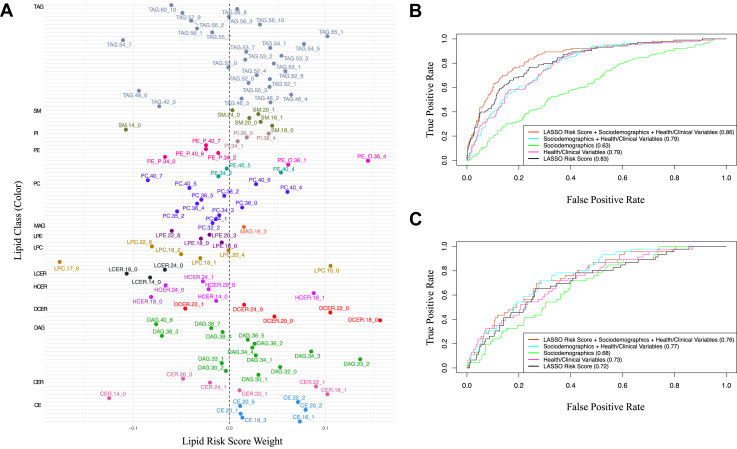
A-C: Weighted LRS derived from penalized (LASSO) regression model. A: Lipid species composition of the LRS. LASSO regression model of lipid species on incident diabetes trained on 0.7 case-balanced data (n cases/noncases = 423/448). LRS comprised LASSO-selected lipid species, weighted by the beta coefficients. Color (*y*-axis) indicates lipid class. *X*-axis is the beta coefficient (weight) of the lipid species in the LRS. B: AUC of the LRS in the 0.7 training data. C: AUC of the LRS in the 0.3 training data. For (B) and (C), color indicates model specifications. Black = LRS, pink = health/clinical variables [physical activity score (continuous) + smoking (current yes/no) + diet (food groups, APDQS, total energy) + eGFR (continuous) + BMI (continuous) + hypertension (yes/no) + lipid-lowering medication use (yes/no)], green = sociodemographics [field center (Birmingham, AL; Chicago, IL; Minneapolis, MN; Oakland, CA) + age (continuous) + sex (male/female) + self-reported race (Black/White) + highest education attained (continuous years)], blue = sociodemographics + health/clinical variables, red = sociodemographics + health/clinical variables + LRS. The true positive rate (sensitivity) and false positive rate (1-specificity) is plotted on the *y*-axis and *x*-axis, respectively. AUCs for each model are noted in the figure legend in parentheses. Higher AUCs indicate increased ability of the model in discriminating 15-year incident diabetes cases from noncases. LRS, lipidomic risk score; LASSO, least absolute shrinkage operator; APDQS, a priori diet quality score; AUC, area under the curve; eGFR, estimated glomerular filtration rate.

The LASSO model trained on the 0.7 data selected 108 of the 300 lipid species (Supplemental Fig. S5; Supplemental Table S5). We note that the 108 selected lipid species included at least one species from each class, and most classes contained species that were both positively and negatively weighted in the LRS (Fig. 3A). Of the 108 lipids, 42 overlapped with statistically significant associations with diabetes from individual regression modeling, including positive associations for CER(22:1), DCER(22:0), PE(40:5), many TAGs, and DAGs; and inverse associations for HCER(14:0), LCER(18:0), LCER(24:0), LPC(18:1), and LPC(18:2).

We used the LRS, derived from model beta-coefficients, to evaluate the extent to which the addition of the LRS to sociodemographic, health behavior, and clinical variables improved prediction of incident diabetes. In the training data, the addition of the LRS to a model of sociodemographics, health behaviors, and clinical measures, predicting 15-year risk of incident diabetes, improved the AUC by 9% (0.79 without LRS; 0.86 with LRS) (Fig. 3B). However, this improvement in the AUC did not replicate in the testing data (0.77 without LRS; 0.79 with LRS) (Fig. 3C).

We next examined whether the predictive ability of 15-year risk of diabetes would improve with a LRS derived from the molecular lipid species (n = 756), using the same analytic framework as above. Despite the greater number and specificity of lipid measures in this analysis, the LASSO selected fewer species (n = 96), compared to the analysis of 300 lipid species. Of the 96 selected molecular lipid species, 51 were significant in individual hazards regression models (Supplemental Fig. S6; Supplemental Table S6). Consistent with analysis of 300 lipid species, the LRS included at least one molecular lipid species from each lipid class (Supplemental Fig. S7), and many lipid classes contained molecular species both positively and negatively weighted in the LRS. In the training data, the addition of this LRS to a model of sociodemographics, health behaviors, and clinical measures predicting 15-year risk of incident diabetes improved the AUC by 8% (0.79 without LRS; 0.85 with LRS), while there was no AUC improvement in the testing data (0.77 for both with and without LRS) (Supplemental Fig. S8).

## Discussion

In a US population-based cohort of early middle-aged adults, we identified lipidomic features associated with incident diabetes. These findings contribute to delineating lipid-related pathways of progression to diabetes informing diabetes risk prediction. Specifically, in both individual regressions and LASSO modeling, we identified 42 lipid species that were associated with incident diabetes; including inverse associations for LPC(18:1), LPC(18:2), LCER(18:0), LCER(24:0), and HCER(14:0); and positive associations for many TAG and DAG species, PE(40:5), DCER(22:0), and CER(22:1). Differential expression analysis revealed associations observed among lipid species within several pathways, including those linking phospholipids to neutral glycerolipids, potentially elucidating specific enzymatic targets relevant to diabetes. Our LASSO-derived LRS, which lacked AUC improvement in the testing data, has implications for ongoing efforts in lipidomic risk score development. These data provide unique value to the literature, as studied CARDIA participants were relatively younger, healthier, and more sociodemographically diverse than many other lipidomic studies of incident T2D (8, 15, 17, 20, 24, 25, 26, 27, 28, 29).

Our findings for glycerolipids are consistent with those of other population-based studies that have reported positive associations between TAGs and DAGs and incident diabetes (14, 15, 16, 26, 30, 31). These results align with mechanistic studies. In the glycerolipid/free fatty acid cycle, TAGs can be stored or utilized as energy when broken down into DAGs and, subsequently, to MAGs and FFAs. The breakdown of TAGs into FFAs and DAGs, but not to MAGs, was upregulated in our pathway activity network. Disruptions in the glycerolipid/free fatty acid cycle can impair B-cell glucose-stimulated insulin secretion and/or cause a lipotoxic accumulation of TAGs and DAGs, contributing to diabetes (32, 33). Previous studies suggest that DAGs activate protein kinase C, disrupting insulin signaling (6). Fatty acid composition of glycerolipids may influence their mechanistic roles, as reflected in our PCA results. The top 2 PCs captured that majority of the variability in the plasma lipidome was related to the FA composition of TAGs, which was associated with risk of T2D. In general, PUFAs are considered beneficial for insulin sensitivity through pleiotropic mechanisms, such as reducing systemic inflammation and improving insulin signaling; however, when incorporated into TAGs, or other complex lipids, these effects are less understood (34, 35). We note that, in contrast to established mechanistic links between PUFAs and glycemic control, evidence from human trials linking dietary PUFA intake and diabetes has been mixed (36, 37).

We identified many sphingolipid species associated with incident diabetes including CER (positively), and LCER and HCER (inversely) species. Similar diabetes associations with CERs, LCERs, and HCERs have been noted in other population-based studies, including MIDUS and PREDIMED (17), though CERs were not associated with diabetes in the Dallas Heart Study (21). In a Mendelian randomization study of metabolomics and diabetes, Li *et al.*, reported that sphingolipid pathways were strongly related to diabetes (22). Pathways leading from SMs and HCERs to CERs were upregulated in our differential expression analysis suggesting that imbalances in sphingolipid metabolism may relate to diabetes pathology. In a previous CARDIA study, CER, SM, LPC, and LPE contributed to a metabolomic-based proinflammatory/adiposity score that was prognostic for incident diabetes. In contrast to Murthy, *et al.*, which used a broad untargeted metabolite platform, we used a fully quantified, targeted lipid panel to inform potential clinical use of lipidomic data specifically. Although Murthy *et al.*’s analysis focused on multimetabolite scores predictive of incident diabetes, our analysis focused on individual lipidomic associations with incident diabetes, FA composition-specific associations, and performance of a LRS that does not include other types of metabolites. Finally, our analysis controlled for more confounding factors including dietary intake (38).

CER species are perhaps the most well-studied species of lipids in sphingolipid metabolism, with glycemic control-related mechanisms elucidated in in vitro, animal, and human studies. Excess adipose tissue increases CER biosynthesis, which contributes to diabetes risk through mechanisms related to insulin and glucose metabolism, including CERs inhibition of P13K/AKT (39, 40). In contrast to CERs and SMs, mechanisms linking LCERs and HCERs to diabetes risk are less well understood. Despite this, LCERs and HCERs have been inversely associated with diabetes risk factors in MIDUS (4), and appear to have beneficial effects on glucose metabolism in mouse models (41). Together these studies and our pathway results support investigation into whether individual sphingolipids or imbalances in sphingolipid metabolism are more related to diabetes pathology.

Similar to sphingolipid species, we found divergent associations among glycerophospholipid species, with PE species positively, and LPC species inversely, associated with incident diabetes. The Mendelian randomization study of Li, *et al.*, reported a strong role for glycerophospholipid pathways in diabetes (22). In our data, pathways involved in synthesizing PC and LPE from PEs were downregulated in diabetes. Some evidence supports a beneficial role for PUFA-containing PEs in insulin signaling (16, 20, 42, 43); though, most epidemiologic studies, including ours, report positive associations between PEs and diabetes (11, 12, 14, 24). In contrast to PEs, results for LPCs have been more consistent across studies. Our inverse associations for LPC species and diabetes align with results from mechanistic models (5), as well as with other epidemiologic studies (11, 12, 14, 15, 20, 24, 28). In mechanistic models, LPCs have displayed beneficial effects in regulating blood glucose levels (5). In epidemiologic studies, LPCs, particularly LPC (18:2), have been robustly associated with reduced diabetes risk (11, 12, 14, 15, 20, 24, 28).

Individual modeling approaches do not address correlations among lipids, and correlated lipids with similar physiological effects may be overrepresented in the lipidomic data. Therefore, approaches that consider lipids simultaneously, like penalized regression and PCA, may better represent the lipidome. LRSs derived from penalized regression have been proposed as an approach for improving risk prediction, though our LASSO-derived LRS improved risk prediction in the training data, but not in the test data. The lack of LRS replication in our test set may have stemmed from insufficient power, though we included the split sample in recognition that this method is more robust than multifold cross-validation. A previous study of gestational diabetes found that the addition of LASSO-selected lipids to a base model improved risk prediction, though this was not validated in test data or an external sample (44). Variability across studies with respect to robust LRS prediction may reflect differences in analytical approach, or in the number or set of included lipids (8, 12, 14, 15, 16, 28). This variability in LRS replication warrants further studies comparing LRSs, particularly those that include external validation. Our approach, LASSO regression, accounts for high correlations among lipids which may benefit summary score derivation from lipidomic data, but we note that not all lipids associated with diabetes will be selected. For example, in our study, the lack of improved prediction when using the 756 molecular lipid species over the 300 lipid species is likely due to high correlations among the molecular lipid species data. Though our predictive power in the training data may be limited, these results suggest the addition of molecular lipid species may not contribute to improvements in prediction, but these data may inform mechanisms of diabetes given their increased structural specificity.

Our study has strengths and limitations. The CARDIA study allowed for 15-year follow-up for incident diabetes in a population-based cohort of early middle-aged Black and White race men and women. The CARDIA study collects data on an extensive set of covariates for control of potential confounders. These data allowed us to adjust for habitual dietary intake as major food groups, diet quality, and total energy, which are known to affect FA composition (45). Although the CARDIA dietary assessment is comprehensive, diet data are subject to measurement error, and residual confounding by diet might remain. Hepatic steatosis is correlated with increased DAGs and TAGs, particularly those containing SFAs, and contributes to T2D pathogenesis, though we did not account for hepatic steatosis in our analysis (18). Accounting for hepatic steatosis in our analysis may attenuate results for glycerolipids and T2D, but we do not have reason to expect significant changes in results for other lipids. We note that 51 (27%) of the incident diabetes cases were determined based on hypoglycemic medication use alone, which was not confirmed for diabetes use. However, based on fasting glucose, 2-h glucose, and HbA1c, 183 (98%) of the incident diabetes cases at least met clinical values indicating prediabetes. Lipidomics data were generated using a targeted platform that quantified 756 molecular lipid species across 14 lipid classes, allowing extensive insight into the plasma lipidome. The platform did not assess free FAs (bound and unbound), which may contribute to a more complete understanding of the lipidome. We conducted analyses at multiple levels of lipid classification to increase comparability to the literature, aid interpretation of results, and evaluate differences among levels. Our pathway analysis is innovative, and identifies overall imbalances in lipid metabolism that contribute to diabetes pathology. Our PCA analysis complemented our other analyses, including LASSO and individual regression, which were less able to capture the variability in TAG FA composition. We used the LASSO selection operator to derive an LRS, a popular method that, to our knowledge, has not been applied to a population-based cohort study of incident diabetes, though we recognize our limited power to significantly predict incident diabetes in the test data. Lauber *et al.*, found that a LRS outperformed a polygenic risk score in predicting T2D; however, we were unable to robustly test a polygenetic risk score, due to limited sample with both genetic and lipidomic data. We note that lipidomic data were derived from plasma collected at a single time point, which may contribute to within-person variability, though this method appears sufficient for most lipids and there is no expected systematic variability (46).

In conclusion, our results identified plasma lipidome features and imbalances in lipid pathways that precede diabetes in a population-based cohort, highlighting species within glycerophospholipid and sphingolipid metabolism. Further investigation of the role of glycerophospholipids and sphingolipids in inflammation and glucose metabolism may confirm whether these lipids can serve as reliable biomarkers of diabetes risk, or if enzymes in related pathways hold potential as therapeutic targets. In contrast to the majority of previous reports in older populations, our data have implications for understanding effects of lipids on diabetes in early middle aged adults, when preventative efforts are most relevant.

## Data Availability

CARDIA data are available upon request from the CARDIA Coordinating Center, using guidance provided at http://www.cardia.dopm.uab.edu/publications-2/publications-documents. CARDIA data are also publicly available on the NIH-supported BioLINCC and dbGaP platforms (phs003675.v1.pa).

### Supplemental Data

This article contains supplemental data (47, 48, 49, 50, 51, 52, 53, 54, 55). (https://www.metabolon.com/wp-content/uploads/2023/03/Metabolon-Panel-Complex-Lipids.pdf).

### Meeting Presentation

This work was presented as a poster at the AHA Epidemiology and Prevention/Lifestyle and Cardiometabolic Health 2025 Scientific Sessions. This abstract was published in Circulation 2025; 151(Suppl_1):P3022.

## Conflict of Interest

The authors declare that they have no conflicts of interest with the contents of this article.

## Supplemental Data

Supplemental Material

Technical Supplement for Methods
