# Supplementary material for: Plasma lipidomics and 15-year risk of incident diabetes: a coronary artery risk development in young adults study
Source: J Lipid Res. 2026 Jan 30;67(3):100992. doi: 10.1016/j.jlr.2026.100992 (PMC12955097; doi:10.1016/j.jlr.2026.100992)

Supplemental Material

Supplemental Figure 1. Study sample flowchart


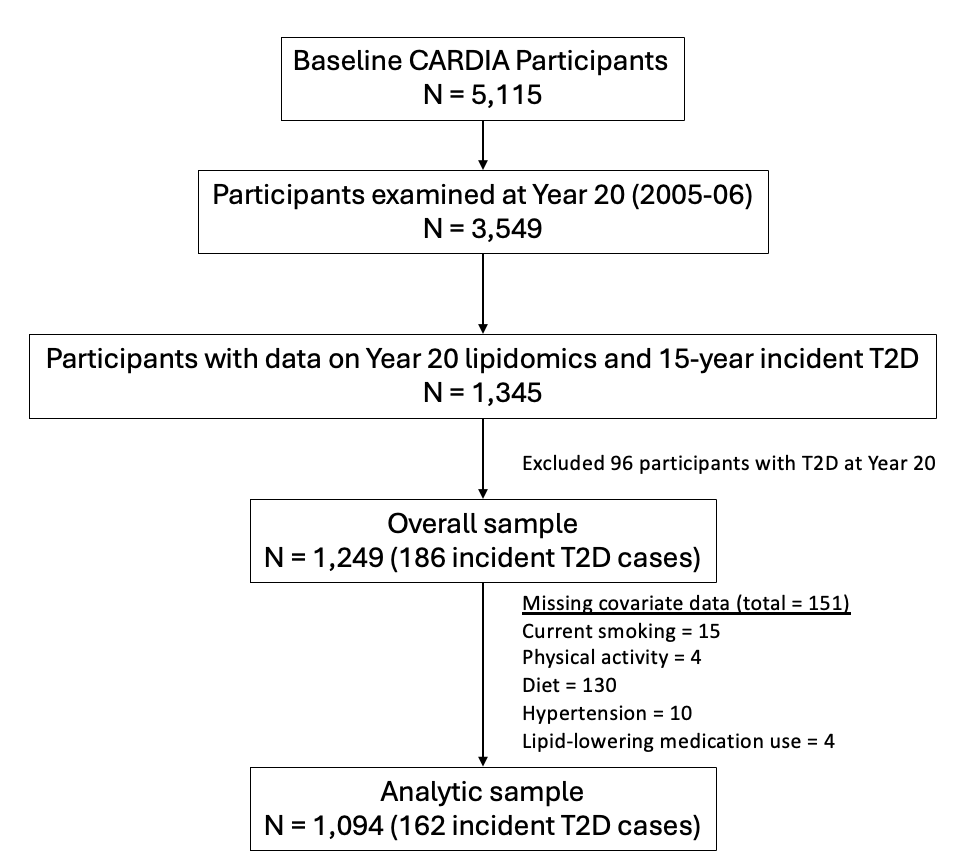


Supplemental Table 1. Medians (IQR) of untransformed lipid class concentrations stratified by 15-year incident diabetes.

|  | 15-Yr Incident Diabetes | |  |
| --- | --- | --- | --- |
| Lipid Class | No | Yes | p-value |
| N | 1,059 | 186 |  |
| Ceramides (uM) | 2.86 (2.44, 3.40) | 3.02 (2.54, 3.55) | 0.07 |
| Cholesteryl ester (uM) | 2,517 (2,246, 2,828) | 2,598 (2,298, 2,934) | 0.07 |
| Diacylglycerol (uM) | 14 (10, 20) | 19 (13, 28) | <0.001 |
| Dihydroceramide (uM) | 0.72 (0.61, 0.87) | 0.80 (0.66, 0.93) | <0.001 |
| Hydrosylceramide (uM) | 2.62 (2.26, 3.00) | 2.46 (2.04, 2.88) | 0.002 |
| Lactosylceramide (uM) | 2.20 (1.92, 2.58) | 1.97 (1.61, 2.37) | <0.001 |
| Lysophosphatidylcholine (uM) | 112 (92, 130) | 95 (81, 116) | <0.001 |
| Lysophosphatidylethanolamine (uM) | 2.50 (2.03, 3.04) | 2.19 (1.82, 2.79) | <0.001 |
| Monoacylglycerol (uM) | 2 (1, 9) | 2 (1, 10) | 0.88 |
| Phosphatidylcholine (uM) | 1,520 (1,350, 1,717) | 1,498 (1,353, 1,731) | 0.89 |
| Phosphatidylethanolamine (uM) | 96 (81, 114) | 98 (83, 117) | 0.13 |
| Phosphatidylinositol (uM) | 17.0 (14.3, 19.9) | 17.0 (14.1, 20.5) | 0.45 |
| Sphingomyelin (uM) | 328 (297, 366) | 328 (295, 360) | 0.78 |
| Triacylglycerol (uM) | 771 (572, 1,117) | 1,108 (774, 1,575) | <0.001 |

Supplemental Table 2. Multivariable-adjusted hazard ratios^1,2^ between lipid classes^3^ and 15-yr incident diabetes^4^

| Lipid Class | Hazard Ratio | Unadjusted p-value | Adjusted p-value |
| --- | --- | --- | --- |
| CER_uM | 1.11 | 2.18E-01 | 5.61E-01 |
| CE_uM | 1.12 | 1.81E-01 | 5.01E-01 |
| DAG_uM | 1.64 | 1.63E-09 | 2.43E-08 |
| DCER_uM | 1.22 | 1.75E-02 | 1.00E-01 |
| HCER_uM | 0.85 | 4.43E-02 | 2.23E-01 |
| LCER_uM | 0.74 | 3.19E-04 | 3.87E-03 |
| LPC_uM | 0.83 | 5.27E-02 | 2.57E-01 |
| LPE_uM | 0.87 | 1.24E-01 | 4.33E-01 |
| MAG_uM | 0.99 | 9.30E-01 | 9.90E-01 |
| PC_uM | 1.13 | 1.40E-01 | 4.61E-01 |
| PE_uM | 1.06 | 5.10E-01 | 7.96E-01 |
| PI_uM | 1.17 | 6.11E-02 | 2.85E-01 |
| SM_uM | 0.89 | 1.60E-01 | 4.75E-01 |
| TAG_uM | 1.74 | 2.37E-11 | 3.93E-10 |

^1^Interval Censored (Weibull) Cox proportional hazards regression model adjusted for sociodemographics (sex, race, age, education), health behaviors (current smoking, physical activity, dietary intake), and clinical variables (BMI, hypertension, lipid-lowering medication use)

^2^Adjusted for multiple comparisons using the false discovery rate (BH)

^3^Lipid classes are log2 transformed and z-scaled

^4^n cases/total = 162/1,094

Supplemental Table 3. Multivariable-adjusted hazard ratios^1,2^ between lipid species^3^ and 15-yr incident diabetes^4^

| Lipid Species | Hazard Ratio | Unadjusted p-value | Adjusted p-value |
| --- | --- | --- | --- |
| CE.12_0 | 1.17 | 5.79E-02 | 2.62E-01 |
| CE.14_0 | 1.27 | 6.69E-03 | 5.18E-02 |
| CE.14_1 | 1.30 | 2.97E-03 | 2.77E-02 |
| CE.15_0 | 1.21 | 3.22E-02 | 1.60E-01 |
| CE.16_0 | 1.15 | 9.40E-02 | 3.61E-01 |
| CE.16_1 | 1.24 | 1.30E-02 | 7.90E-02 |
| CE.17_0 | 1.23 | 1.53E-02 | 8.71E-02 |
| CE.18_0 | 1.20 | 2.76E-02 | 1.40E-01 |
| CE.18_1 | 1.04 | 6.57E-01 | 9.20E-01 |
| CE.18_2 | 1.08 | 3.67E-01 | 6.79E-01 |
| CE.18_3 | 1.12 | 1.77E-01 | 4.99E-01 |
| CE.18_4 | 1.10 | 2.71E-01 | 6.18E-01 |
| CE.20_0 | 1.15 | 3.21E-02 | 1.60E-01 |
| CE.20_1 | 1.10 | 1.39E-01 | 4.53E-01 |
| CE.20_2 | 1.22 | 1.88E-02 | 1.01E-01 |
| CE.20_3 | 1.27 | 6.85E-03 | 5.25E-02 |
| CE.20_4 | 1.04 | 6.69E-01 | 9.29E-01 |
| CE.20_5 | 1.05 | 6.13E-01 | 8.87E-01 |
| CE.22_0 | 1.03 | 7.06E-01 | 9.48E-01 |
| CE.22_1 | 1.02 | 8.07E-01 | 9.81E-01 |
| CE.22_2 | 1.17 | 1.51E-02 | 8.66E-02 |
| CE.22_4 | 1.15 | 1.13E-01 | 4.04E-01 |
| CE.22_5 | 1.18 | 6.49E-02 | 2.83E-01 |
| CE.22_6 | 1.03 | 7.82E-01 | 9.72E-01 |
| CE.24_0 | 1.05 | 5.29E-01 | 8.26E-01 |
| CE.24_1 | 1.05 | 5.53E-01 | 8.50E-01 |
| CER.14_0 | 0.99 | 9.41E-01 | 9.93E-01 |
| CER.16_0 | 1.08 | 3.61E-01 | 6.79E-01 |
| CER.18_0 | 1.40 | 1.08E-04 | 1.47E-03 |
| CER.18_1 | 1.30 | 9.01E-03 | 6.02E-02 |
| CER.20_0 | 1.25 | 6.88E-03 | 5.25E-02 |
| CER.20_1 | 1.28 | 4.21E-02 | 2.01E-01 |
| CER.22_0 | 1.21 | 2.86E-02 | 1.44E-01 |
| CER.22_1 | 1.34 | 8.75E-04 | 8.69E-03 |
| CER.24_0 | 1.02 | 8.48E-01 | 9.92E-01 |
| CER.24_1 | 1.17 | 6.51E-02 | 2.83E-01 |
| CER.26_0 | 0.95 | 5.78E-01 | 8.65E-01 |
| CER.26_1 | 1.04 | 6.26E-01 | 8.98E-01 |
| DAG.30_0 | 1.19 | 9.66E-02 | 3.66E-01 |
| DAG.30_1 | 1.25 | 1.34E-02 | 8.04E-02 |
| DAG.30_2 | 1.25 | 1.46E-02 | 8.54E-02 |
| DAG.32_0 | 1.32 | 1.45E-02 | 8.47E-02 |
| DAG.32_1 | 1.76 | 5.98E-10 | 9.37E-09 |
| DAG.32_2 | 1.73 | 2.51E-08 | 3.74E-07 |
| DAG.32_3 | 1.29 | 1.00E-02 | 6.48E-02 |
| DAG.33_1 | 1.65 | 3.80E-06 | 5.37E-05 |
| DAG.33_2 | 1.25 | 8.99E-03 | 6.02E-02 |
| DAG.34_0 | 1.33 | 3.57E-03 | 3.30E-02 |
| DAG.34_1 | 1.69 | 1.83E-10 | 2.89E-09 |
| DAG.34_2 | 1.77 | 1.00E-11 | 1.67E-10 |
| DAG.34_3 | 1.76 | 1.34E-11 | 2.21E-10 |
| DAG.34_4 | 1.68 | 5.11E-07 | 7.38E-06 |
| DAG.36_1 | 1.62 | 1.05E-09 | 1.63E-08 |
| DAG.36_2 | 1.70 | 1.15E-10 | 1.83E-09 |
| DAG.36_3 | 1.65 | 1.45E-09 | 2.23E-08 |
| DAG.36_4 | 1.48 | 7.22E-07 | 1.04E-05 |
| DAG.36_5 | 1.59 | 9.63E-07 | 1.38E-05 |
| DAG.36_6 | 1.13 | 1.84E-01 | 5.12E-01 |
| DAG.38_2 | 1.53 | 5.98E-08 | 8.84E-07 |
| DAG.38_3 | 1.42 | 2.38E-05 | 3.35E-04 |
| DAG.38_4 | 1.41 | 4.17E-05 | 5.84E-04 |
| DAG.38_5 | 1.58 | 5.12E-08 | 7.61E-07 |
| DAG.38_6 | 1.53 | 1.08E-06 | 1.54E-05 |
| DAG.38_7 | 1.48 | 1.60E-04 | 2.07E-03 |
| DAG.40_5 | 1.53 | 6.57E-07 | 9.49E-06 |
| DAG.40_6 | 1.13 | 1.76E-01 | 4.97E-01 |
| DAG.40_7 | 1.54 | 1.21E-06 | 1.73E-05 |
| DAG.40_8 | 1.40 | 4.31E-04 | 5.05E-03 |
| DCER.16_0 | 1.07 | 3.98E-01 | 6.96E-01 |
| DCER.18_0 | 1.26 | 1.06E-02 | 6.75E-02 |
| DCER.20_0 | 1.11 | 2.32E-01 | 5.76E-01 |
| DCER.22_0 | 1.35 | 5.71E-04 | 6.20E-03 |
| DCER.22_1 | 1.07 | 4.94E-01 | 7.90E-01 |
| DCER.22_2 | 1.38 | 1.05E-03 | 1.03E-02 |
| DCER.24_0 | 1.08 | 4.04E-01 | 7.02E-01 |
| DCER.24_1 | 1.25 | 1.14E-02 | 7.13E-02 |
| HCER.14_0 | 0.77 | 6.06E-04 | 6.46E-03 |
| HCER.16_0 | 0.89 | 1.78E-01 | 5.00E-01 |
| HCER.18_0 | 0.88 | 1.34E-01 | 4.45E-01 |
| HCER.18_1 | 1.00 | 9.78E-01 | 9.97E-01 |
| HCER.20_0 | 0.91 | 2.68E-01 | 6.14E-01 |
| HCER.22_0 | 0.87 | 8.00E-02 | 3.24E-01 |
| HCER.22_1 | 0.99 | 8.97E-01 | 9.93E-01 |
| HCER.24_0 | 0.84 | 3.26E-02 | 1.62E-01 |
| HCER.24_1 | 0.84 | 3.86E-02 | 1.86E-01 |
| LCER.14_0 | 0.86 | 4.15E-02 | 1.98E-01 |
| LCER.16_0 | 0.76 | 9.10E-04 | 9.03E-03 |
| LCER.18_0 | 0.76 | 2.75E-03 | 2.57E-02 |
| LCER.18_1 | 0.87 | 5.07E-02 | 2.37E-01 |
| LCER.20_0 | 0.96 | 6.02E-01 | 8.79E-01 |
| LCER.22_0 | 0.82 | 1.85E-02 | 9.97E-02 |
| LCER.22_1 | 0.93 | 3.23E-01 | 6.53E-01 |
| LCER.24_0 | 0.73 | 4.89E-04 | 5.59E-03 |
| LCER.24_1 | 0.71 | 6.09E-05 | 8.46E-04 |
| LPC.15_0 | 1.16 | 8.74E-02 | 3.44E-01 |
| LPC.16_0 | 0.91 | 2.82E-01 | 6.23E-01 |
| LPC.16_1 | 0.99 | 8.90E-01 | 9.93E-01 |
| LPC.17_0 | 0.85 | 5.85E-02 | 2.64E-01 |
| LPC.18_0 | 0.90 | 2.09E-01 | 5.53E-01 |
| LPC.18_1 | 0.74 | 1.78E-03 | 1.69E-02 |
| LPC.18_2 | 0.69 | 1.95E-04 | 2.49E-03 |
| LPC.20_2 | 0.95 | 5.17E-01 | 8.14E-01 |
| LPC.20_3 | 0.95 | 5.52E-01 | 8.50E-01 |
| LPC.20_4 | 0.80 | 1.33E-02 | 8.00E-02 |
| LPC.22_6 | 0.90 | 1.87E-01 | 5.17E-01 |
| LPE.16_0 | 0.86 | 8.84E-02 | 3.46E-01 |
| LPE.18_0 | 0.91 | 2.93E-01 | 6.30E-01 |
| LPE.18_1 | 0.90 | 2.40E-01 | 5.83E-01 |
| LPE.18_2 | 0.88 | 1.45E-01 | 4.63E-01 |
| LPE.20_3 | 0.98 | 8.16E-01 | 9.84E-01 |
| LPE.20_4 | 0.86 | 1.29E-01 | 4.36E-01 |
| LPE.22_5 | 1.02 | 7.92E-01 | 9.76E-01 |
| LPE.22_6 | 0.85 | 7.13E-02 | 2.99E-01 |
| MAG.14_1 | 0.98 | 8.14E-01 | 9.83E-01 |
| MAG.16_1 | 1.28 | 2.02E-02 | 1.07E-01 |
| MAG.18_1 | 1.12 | 2.28E-01 | 5.70E-01 |
| MAG.18_2 | 1.41 | 6.11E-04 | 6.47E-03 |
| MAG.18_3 | 1.15 | 1.11E-01 | 3.99E-01 |
| MAG.20_0 | 1.04 | 6.54E-01 | 9.17E-01 |
| PC.30_0 | 1.13 | 1.39E-01 | 4.52E-01 |
| PC.32_0 | 1.04 | 6.38E-01 | 9.07E-01 |
| PC.32_1 | 1.17 | 6.84E-02 | 2.90E-01 |
| PC.32_2 | 1.07 | 4.84E-01 | 7.81E-01 |
| PC.33_2 | 1.16 | 9.79E-02 | 3.70E-01 |
| PC.34_0 | 1.01 | 9.03E-01 | 9.93E-01 |
| PC.34_1 | 1.15 | 1.03E-01 | 3.81E-01 |
| PC.34_2 | 1.04 | 6.22E-01 | 8.95E-01 |
| PC.34_3 | 1.06 | 4.82E-01 | 7.79E-01 |
| PC.34_4 | 1.13 | 1.90E-01 | 5.21E-01 |
| PC.35_1 | 1.08 | 3.54E-01 | 6.74E-01 |
| PC.35_2 | 1.03 | 7.24E-01 | 9.54E-01 |
| PC.36_0 | 1.18 | 4.86E-02 | 2.28E-01 |
| PC.36_1 | 0.94 | 4.33E-01 | 7.27E-01 |
| PC.36_2 | 1.09 | 3.01E-01 | 6.34E-01 |
| PC.36_3 | 1.17 | 7.09E-02 | 2.98E-01 |
| PC.36_4 | 0.98 | 7.81E-01 | 9.72E-01 |
| PC.36_5 | 1.12 | 1.67E-01 | 4.83E-01 |
| PC.37_4 | 1.02 | 8.64E-01 | 9.93E-01 |
| PC.38_2 | 1.08 | 3.55E-01 | 6.74E-01 |
| PC.38_3 | 1.20 | 4.47E-02 | 2.12E-01 |
| PC.38_4 | 1.21 | 3.76E-02 | 1.82E-01 |
| PC.38_5 | 0.98 | 8.04E-01 | 9.81E-01 |
| PC.38_6 | 1.01 | 9.07E-01 | 9.93E-01 |
| PC.40_4 | 1.25 | 1.91E-02 | 1.02E-01 |
| PC.40_5 | 1.20 | 2.83E-02 | 1.43E-01 |
| PC.40_6 | 1.07 | 4.02E-01 | 7.01E-01 |
| PC.40_7 | 0.92 | 3.03E-01 | 6.36E-01 |
| PE.34_1 | 1.35 | 9.43E-04 | 9.32E-03 |
| PE.34_2 | 1.24 | 9.87E-03 | 6.42E-02 |
| PE.34_3 | 1.02 | 8.47E-01 | 9.92E-01 |
| PE.36_0 | 1.01 | 9.61E-01 | 9.94E-01 |
| PE.36_1 | 1.11 | 2.43E-01 | 5.86E-01 |
| PE.36_2 | 1.28 | 3.25E-03 | 3.03E-02 |
| PE.36_3 | 1.21 | 2.19E-02 | 1.15E-01 |
| PE.36_4 | 1.21 | 2.90E-02 | 1.46E-01 |
| PE.38_2 | 0.96 | 6.09E-01 | 8.84E-01 |
| PE.38_3 | 1.32 | 1.58E-03 | 1.51E-02 |
| PE.38_4 | 1.27 | 4.62E-03 | 4.14E-02 |
| PE.38_5 | 1.28 | 7.38E-03 | 5.46E-02 |
| PE.38_6 | 1.17 | 1.06E-01 | 3.86E-01 |
| PE.40_4 | 1.10 | 2.95E-01 | 6.30E-01 |
| PE.40_5 | 1.34 | 5.85E-04 | 6.29E-03 |
| PE.40_6 | 1.22 | 3.69E-02 | 1.79E-01 |
| PE_O.36_1 | 1.13 | 1.35E-01 | 4.46E-01 |
| PE_O.36_2 | 0.99 | 9.39E-01 | 9.93E-01 |
| PE_O.36_4 | 1.15 | 1.03E-01 | 3.80E-01 |
| PE_O.38_4 | 0.98 | 8.13E-01 | 9.83E-01 |
| PE_O.38_5 | 0.95 | 5.60E-01 | 8.55E-01 |
| PE_P.34_0 | 0.96 | 5.94E-01 | 8.76E-01 |
| PE_P.34_1 | 0.85 | 8.46E-02 | 3.37E-01 |
| PE_P.34_2 | 0.88 | 1.16E-01 | 4.11E-01 |
| PE_P.36_1 | 0.87 | 8.93E-02 | 3.49E-01 |
| PE_P.36_2 | 0.85 | 4.07E-02 | 1.95E-01 |
| PE_P.36_3 | 0.97 | 7.00E-01 | 9.47E-01 |
| PE_P.36_4 | 0.97 | 7.04E-01 | 9.47E-01 |
| PE_P.38_3 | 0.94 | 4.98E-01 | 7.95E-01 |
| PE_P.38_4 | 0.95 | 5.56E-01 | 8.53E-01 |
| PE_P.38_5 | 0.95 | 5.57E-01 | 8.53E-01 |
| PE_P.38_6 | 0.94 | 4.82E-01 | 7.79E-01 |
| PE_P.40_4 | 0.91 | 2.76E-01 | 6.20E-01 |
| PE_P.40_5 | 0.88 | 1.32E-01 | 4.41E-01 |
| PE_P.40_6 | 0.83 | 1.40E-02 | 8.28E-02 |
| PE_P.40_7 | 0.86 | 7.34E-02 | 3.06E-01 |
| PI.34_1 | 1.10 | 2.89E-01 | 6.28E-01 |
| PI.34_2 | 1.18 | 5.96E-02 | 2.69E-01 |
| PI.36_1 | 1.11 | 2.25E-01 | 5.67E-01 |
| PI.36_2 | 1.04 | 6.28E-01 | 8.99E-01 |
| PI.36_3 | 1.07 | 4.75E-01 | 7.72E-01 |
| PI.36_4 | 1.12 | 1.90E-01 | 5.21E-01 |
| PI.38_3 | 1.17 | 7.36E-02 | 3.06E-01 |
| PI.38_4 | 1.10 | 2.40E-01 | 5.82E-01 |
| PI.38_5 | 1.05 | 5.97E-01 | 8.76E-01 |
| SM.14_0 | 1.01 | 9.31E-01 | 9.93E-01 |
| SM.16_0 | 0.81 | 1.24E-02 | 7.57E-02 |
| SM.18_0 | 1.13 | 1.44E-01 | 4.61E-01 |
| SM.18_1 | 1.05 | 6.24E-01 | 8.97E-01 |
| SM.20_0 | 1.06 | 4.86E-01 | 7.83E-01 |
| SM.20_1 | 0.91 | 2.96E-01 | 6.30E-01 |
| SM.22_0 | 1.03 | 7.04E-01 | 9.47E-01 |
| SM.22_1 | 0.84 | 4.29E-02 | 2.04E-01 |
| SM.24_0 | 0.92 | 2.97E-01 | 6.30E-01 |
| SM.24_1 | 0.79 | 7.07E-03 | 5.33E-02 |
| SM.26_0 | 0.98 | 7.63E-01 | 9.67E-01 |
| SM.26_1 | 0.90 | 1.75E-01 | 4.95E-01 |
| TAG.42_0 | 1.25 | 1.53E-02 | 8.71E-02 |
| TAG.42_1 | 1.29 | 4.97E-03 | 4.39E-02 |
| TAG.44_0 | 1.37 | 1.13E-03 | 1.09E-02 |
| TAG.44_1 | 1.43 | 1.27E-04 | 1.70E-03 |
| TAG.44_2 | 1.41 | 8.37E-05 | 1.16E-03 |
| TAG.44_3 | 1.44 | 6.39E-05 | 8.87E-04 |
| TAG.45_0 | 1.35 | 3.23E-03 | 3.00E-02 |
| TAG.45_1 | 1.15 | 1.48E-01 | 4.65E-01 |
| TAG.46_0 | 1.44 | 1.17E-04 | 1.58E-03 |
| TAG.46_1 | 1.54 | 7.74E-07 | 1.11E-05 |
| TAG.46_2 | 1.59 | 4.09E-07 | 5.93E-06 |
| TAG.46_3 | 1.56 | 1.82E-06 | 2.58E-05 |
| TAG.46_4 | 1.49 | 5.13E-06 | 7.25E-05 |
| TAG.47_0 | 1.50 | 4.41E-05 | 6.15E-04 |
| TAG.47_1 | 1.60 | 4.44E-05 | 6.17E-04 |
| TAG.47_2 | 1.55 | 3.03E-05 | 4.26E-04 |
| TAG.48_0 | 1.58 | 4.37E-07 | 6.33E-06 |
| TAG.48_1 | 1.66 | 1.69E-07 | 2.48E-06 |
| TAG.48_2 | 1.63 | 7.76E-08 | 1.15E-06 |
| TAG.48_3 | 1.66 | 2.07E-08 | 3.10E-07 |
| TAG.48_4 | 1.67 | 5.92E-08 | 8.78E-07 |
| TAG.48_5 | 1.41 | 5.70E-04 | 6.20E-03 |
| TAG.49_0 | 1.59 | 1.57E-06 | 2.23E-05 |
| TAG.49_1 | 1.73 | 1.01E-08 | 1.52E-07 |
| TAG.49_2 | 1.75 | 3.00E-09 | 4.60E-08 |
| TAG.49_3 | 1.76 | 6.27E-09 | 9.52E-08 |
| TAG.50_0 | 1.62 | 3.48E-07 | 5.06E-06 |
| TAG.50_1 | 1.66 | 9.17E-09 | 1.39E-07 |
| TAG.50_2 | 1.69 | 4.06E-09 | 6.20E-08 |
| TAG.50_3 | 1.75 | 5.17E-10 | 8.13E-09 |
| TAG.50_4 | 1.75 | 1.20E-09 | 1.85E-08 |
| TAG.50_5 | 1.69 | 8.13E-08 | 1.20E-06 |
| TAG.51_0 | 1.66 | 2.03E-07 | 2.96E-06 |
| TAG.51_1 | 1.68 | 8.28E-10 | 1.29E-08 |
| TAG.51_2 | 1.77 | 8.19E-11 | 1.31E-09 |
| TAG.51_3 | 1.85 | 5.79E-11 | 9.31E-10 |
| TAG.51_4 | 1.77 | 7.38E-09 | 1.12E-07 |
| TAG.51_5 | 1.56 | 8.62E-06 | 1.22E-04 |
| TAG.52_0 | 1.61 | 1.80E-08 | 2.70E-07 |
| TAG.52_1 | 1.68 | 9.68E-10 | 1.51E-08 |
| TAG.52_2 | 1.75 | 1.66E-10 | 2.63E-09 |
| TAG.52_3 | 1.75 | 1.67E-10 | 2.65E-09 |
| TAG.52_4 | 1.73 | 7.54E-10 | 1.18E-08 |
| TAG.52_5 | 1.74 | 1.02E-09 | 1.59E-08 |
| TAG.52_6 | 1.66 | 4.14E-08 | 6.16E-07 |
| TAG.52_7 | 1.60 | 1.52E-06 | 2.17E-05 |
| TAG.52_8 | 1.75 | 3.83E-07 | 5.57E-06 |
| TAG.53_0 | 1.60 | 2.57E-07 | 3.75E-06 |
| TAG.53_1 | 1.62 | 3.44E-09 | 5.26E-08 |
| TAG.53_2 | 1.70 | 1.63E-10 | 2.60E-09 |
| TAG.53_3 | 1.71 | 3.56E-09 | 5.43E-08 |
| TAG.53_4 | 1.68 | 2.07E-08 | 3.10E-07 |
| TAG.53_5 | 1.83 | 5.56E-08 | 8.25E-07 |
| TAG.53_6 | 1.75 | 4.22E-05 | 5.90E-04 |
| TAG.53_7 | 1.51 | 5.42E-04 | 5.99E-03 |
| TAG.54_0 | 1.48 | 3.61E-05 | 5.06E-04 |
| TAG.54_1 | 1.58 | 6.05E-09 | 9.21E-08 |
| TAG.54_2 | 1.66 | 3.02E-10 | 4.75E-09 |
| TAG.54_3 | 1.70 | 1.19E-10 | 1.90E-09 |
| TAG.54_4 | 1.71 | 1.95E-10 | 3.08E-09 |
| TAG.54_5 | 1.69 | 1.08E-09 | 1.67E-08 |
| TAG.54_6 | 1.69 | 2.01E-09 | 3.09E-08 |
| TAG.54_7 | 1.61 | 1.63E-07 | 2.38E-06 |
| TAG.54_8 | 1.46 | 4.43E-05 | 6.17E-04 |
| TAG.55_1 | 1.57 | 6.69E-07 | 9.64E-06 |
| TAG.55_2 | 1.49 | 2.63E-07 | 3.83E-06 |
| TAG.55_3 | 1.58 | 5.89E-10 | 9.24E-09 |
| TAG.55_4 | 1.60 | 1.96E-10 | 3.09E-09 |
| TAG.55_5 | 1.63 | 7.04E-10 | 1.10E-08 |
| TAG.55_6 | 1.72 | 1.46E-07 | 2.15E-06 |
| TAG.55_7 | 1.51 | 3.30E-05 | 4.64E-04 |
| TAG.55_8 | 1.22 | 3.30E-02 | 1.63E-01 |
| TAG.56_1 | 1.21 | 7.22E-02 | 3.02E-01 |
| TAG.56_10 | 1.61 | 3.45E-08 | 5.14E-07 |
| TAG.56_2 | 1.45 | 9.59E-06 | 1.35E-04 |
| TAG.56_3 | 1.55 | 1.74E-08 | 2.62E-07 |
| TAG.56_4 | 1.57 | 1.55E-08 | 2.33E-07 |
| TAG.56_5 | 1.58 | 2.21E-08 | 3.31E-07 |
| TAG.56_6 | 1.61 | 1.40E-08 | 2.11E-07 |
| TAG.56_7 | 1.60 | 8.24E-08 | 1.21E-06 |
| TAG.56_8 | 1.54 | 2.06E-06 | 2.92E-05 |
| TAG.56_9 | 1.34 | 1.22E-03 | 1.18E-02 |
| TAG.57_2 | 1.33 | 1.50E-03 | 1.44E-02 |
| TAG.57_3 | 1.19 | 6.49E-02 | 2.83E-01 |
| TAG.57_8 | 1.39 | 2.54E-03 | 2.39E-02 |
| TAG.57_9 | 1.52 | 2.97E-03 | 2.77E-02 |
| TAG.58_10 | 1.20 | 5.37E-02 | 2.48E-01 |
| TAG.58_3 | 1.20 | 5.53E-02 | 2.54E-01 |
| TAG.58_5 | 1.56 | 1.81E-06 | 2.57E-05 |
| TAG.58_6 | 1.53 | 1.79E-06 | 2.55E-05 |
| TAG.58_7 | 1.58 | 1.26E-07 | 1.85E-06 |
| TAG.58_8 | 1.41 | 1.01E-04 | 1.38E-03 |
| TAG.58_9 | 1.34 | 9.60E-04 | 9.45E-03 |
| TAG.60_10 | 1.16 | 1.13E-01 | 4.03E-01 |
| TAG.60_11 | 1.18 | 1.19E-01 | 4.16E-01 |
| TAG.60_12 | 1.08 | 4.25E-01 | 7.22E-01 |

^1^Interval Censored Cox (Weibull) proportional hazards regression model adjusted for sociodemographics (sex, race, age, education), health behaviors (current smoking, physical activity, dietary intake), and clinical variables (BMI, hypertension, lipid-lowering medication use)

^2^Adjusted for multiple comparisons using the false discovery rate (BH)

^3^Lipid species are molecular lipid species grouped by total carbon and total double bond counts, log2 transformed and z-scaled

^4^n cases/total = 162/1,094

Supplemental Figure 2A-B. Differential expressed lipid species in participants who did or did not develop diabetes over the 15-year study period. A) Volcano plot of lipid species altered in incident diabetes cases. Red and blue dots indicate lipid species increased and decreased in incident diabetes cases, respectively. Grey dots indicate lipid species that were not differentially expressed between incident diabetes cases and non-cases. B) Lollipop plot of the top 10 increased and top 10 decreased lipid species in incident diabetes cases compared to non-cases, based on the magnitude of fold change. Color of dots indicates strength of association (darker = more significant, lighter = less significant).


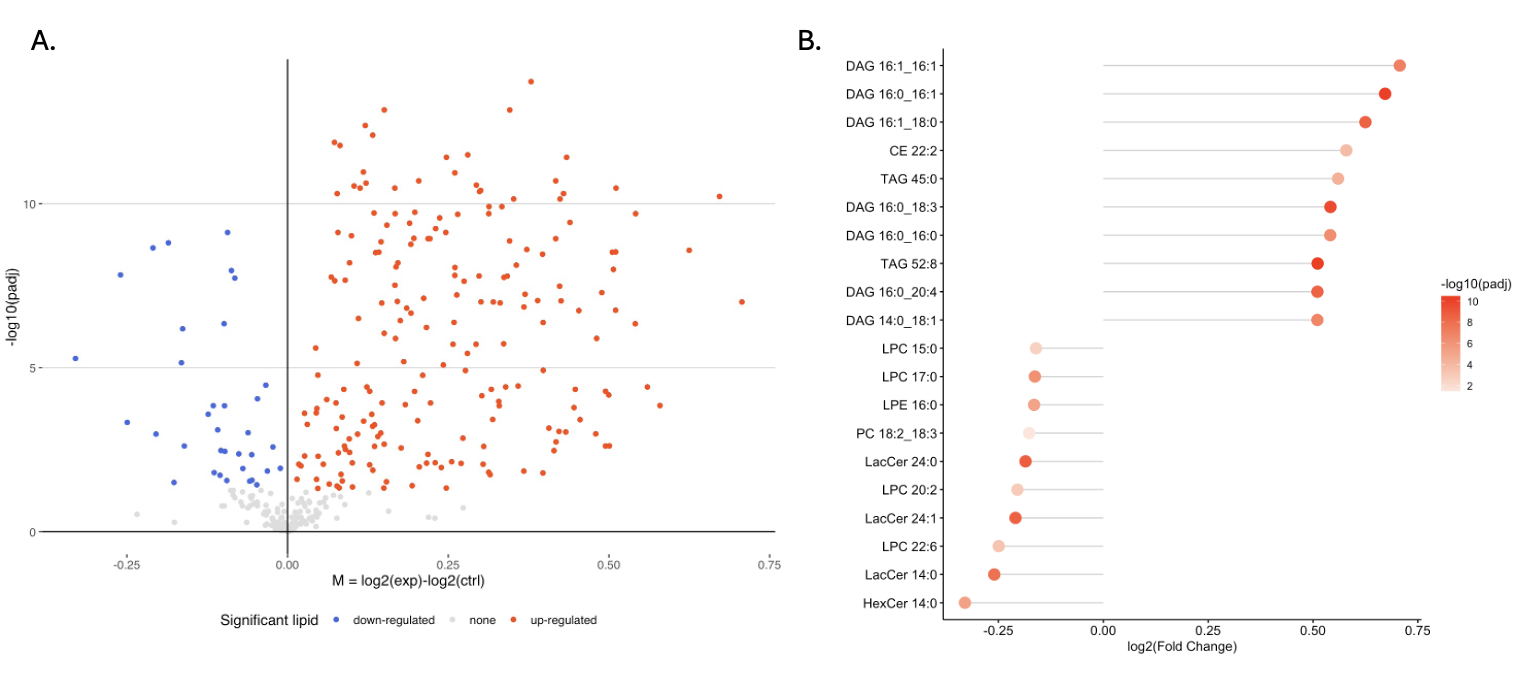


Supplemental Table 4. Summary table of differentially expressed lipids in participants who developed incident diabetes. Counts are the number of lipid species differentially expressed in each lipid class grouping.

| Lipid Class | Count (%) |
| --- | --- |
| DAG | 49 (21%) |
| TAG | 95 (41%) |
| CER | 8 (3.4%) |
| LPC | 9 (3.8%) |
| LCER | 6 (2.6%) |
| Hex2Cer | 5 (2.1%) |
| HCER | 6 (2.6%) |
| CE | 14 (6.0%) |
| PC | 18 (7.7%) |
| MAG | 4 (1.7%) |
| SM | 3 (1.3%) |
| LPE | 3 (1.3%) |
| PI | 3 (1.3%) |
| PE-P | 2 (0.9%) |
| PE | 9 (3.8%) |
| Total | 234 (100%) |

Supplemental Figure 3. Scree plot displaying the proportion of variance explained by orthogonal principal components generated from principal components analysis of 756 molecular lipid species.

Supplemental Figure 4A-B. Top molecular lipid species loadings for principal component 1 and principal component 2 from principal components analysis of the 756 molecular lipid species. A) Principal component 1 explained 46.2% of the lipidome variability. B) Principal component 2 explained 6.5% of the lipidome variability. Loadings shown are those with an absolute value > 0.06.

Supplemental Figure 5. Volcano plot of associations between lipid species and incident diabetes in individual interval censored (Weibull) Cox proportional hazards regression models adjusted for sociodemographics, health behaviors, and clinical variables. Hazard ratios are on the x-axis. Transformed (-log10) adjusted (FDR) p-values are on the y-axis. Top right quadrant = positively associated, top left quadrant = inversely associated, bottom left and right quadrants = insignificant. Red color with labels indicate lipid species that were significant in individual regression and selected by the LASSO penalty in simultaneous penalized regression modeling. Penalized (LASSO) regression model trained on case-balanced 0.7 data with 10 fold cross-validation. Lipid species are molecular lipid species grouped by total carbon and total double bond counts, log2 transformed and z-scaled.

Supplemental table 5. Lipid species^1^ significantly associated^2^ with incident diabetes in individual regression^3^ (n=130 lipids), penalized (LASSO) regression^4^ (n=108 lipids), or both (n=42 lipids).

| Lipid Species | LASSO Regression Beta Coefficient | Hazard ratio from individual regression models | Significant in both individual and LASSO regression models |
| --- | --- | --- | --- |
| CER.22_1 | 0.0901338 | 1.335 | 1 |
| DAG.33_1 | -0.0079048 | 1.653 | 1 |
| DAG.34_1 | 0.0274194 | 1.687 | 1 |
| DAG.34_3 | 0.08503514 | 1.761 | 1 |
| DAG.34_4 | 0.02381388 | 1.68 | 1 |
| DAG.36_2 | 0.02627771 | 1.695 | 1 |
| DAG.36_5 | 0.01904327 | 1.59 | 1 |
| DAG.38_3 | -0.0702587 | 1.417 | 1 |
| DAG.38_4 | -0.0070912 | 1.409 | 1 |
| DAG.38_7 | -0.0189352 | 1.478 | 1 |
| DAG.40_8 | -0.0761746 | 1.401 | 1 |
| DCER.22_0 | 0.10497924 | 1.348 | 1 |
| HCER.14_0 | -0.0137367 | 0.7729 | 1 |
| LCER.18_0 | -0.1068575 | 0.7634 | 1 |
| LCER.24_0 | -0.0671267 | 0.7331 | 1 |
| LPC.18_1 | -0.0303116 | 0.7423 | 1 |
| LPC.18_2 | -0.050306 | 0.6869 | 1 |
| PE.40_5 | -0.0027929 | 1.335 | 1 |
| TAG.46_2 | 0.04272266 | 1.59 | 1 |
| TAG.46_3 | 0.01265777 | 1.562 | 1 |
| TAG.46_4 | 0.06427699 | 1.492 | 1 |
| TAG.48_0 | -0.0941754 | 1.583 | 1 |
| TAG.50_3 | 0.04173943 | 1.748 | 1 |
| TAG.52_0 | 0.01983558 | 1.607 | 1 |
| TAG.52_1 | 0.0420683 | 1.675 | 1 |
| TAG.52_4 | 0.028436 | 1.73 | 1 |
| TAG.52_8 | 0.05819251 | 1.752 | 1 |
| TAG.53_0 | -0.0011161 | 1.596 | 1 |
| TAG.53_1 | 0.04615224 | 1.622 | 1 |
| TAG.53_2 | 0.01651706 | 1.695 | 1 |
| TAG.53_3 | 0.05411427 | 1.711 | 1 |
| TAG.53_7 | 0.01750725 | 1.511 | 1 |
| TAG.54_1 | 0.04244296 | 1.577 | 1 |
| TAG.54_5 | 0.07751403 | 1.69 | 1 |
| TAG.54_7 | -0.1107158 | 1.612 | 1 |
| TAG.55_1 | 0.10194916 | 1.567 | 1 |
| TAG.55_7 | -0.0178568 | 1.51 | 1 |
| TAG.56_10 | 0.03167419 | 1.611 | 1 |
| TAG.56_2 | -0.0398415 | 1.445 | 1 |
| TAG.56_3 | -0.0006081 | 1.545 | 1 |
| TAG.57_9 | -0.0488998 | 1.517 | 1 |
| TAG.58_8 | 0.0081182 | 1.405 | 1 |
| CE.14_1 | NA | 1.303 | 0 |
| CE.16_1 | 0.07323447 | NA | 0 |
| CE.18_3 | 0.01347566 | NA | 0 |
| CE.20_1 | 0.01164417 | NA | 0 |
| CE.20_2 | 0.07928355 | NA | 0 |
| CE.20_5 | 0.01113918 | NA | 0 |
| CE.22_2 | 0.0713044 | NA | 0 |
| CER.14_0 | -0.125095 | NA | 0 |
| CER.18_0 | NA | 1.404 | 0 |
| CER.18_1 | 0.10188273 | NA | 0 |
| CER.20_1 | 0.01048719 | NA | 0 |
| CER.24_1 | -0.0201934 | NA | 0 |
| CER.26_0 | -0.0482156 | NA | 0 |
| DAG.30_1 | 0.03021675 | NA | 0 |
| DAG.30_2 | -0.003632 | NA | 0 |
| DAG.32_0 | 0.05271949 | NA | 0 |
| DAG.32_1 | NA | 1.758 | 0 |
| DAG.32_2 | NA | 1.733 | 0 |
| DAG.33_2 | 0.13602971 | NA | 0 |
| DAG.34_0 | NA | 1.331 | 0 |
| DAG.34_2 | NA | 1.774 | 0 |
| DAG.36_1 | NA | 1.623 | 0 |
| DAG.36_3 | NA | 1.648 | 0 |
| DAG.36_4 | NA | 1.484 | 0 |
| DAG.38_2 | NA | 1.533 | 0 |
| DAG.38_5 | NA | 1.575 | 0 |
| DAG.38_6 | NA | 1.532 | 0 |
| DAG.40_5 | NA | 1.526 | 0 |
| DAG.40_7 | NA | 1.543 | 0 |
| DCER.18_0 | 0.15670985 | NA | 0 |
| DCER.20_0 | 0.04680529 | NA | 0 |
| DCER.22_1 | -0.0459569 | NA | 0 |
| DCER.22_2 | NA | 1.381 | 0 |
| DCER.24_0 | 0.01528293 | NA | 0 |
| HCER.18_0 | -0.0814446 | NA | 0 |
| HCER.18_1 | 0.08767291 | NA | 0 |
| HCER.20_0 | -0.0216234 | NA | 0 |
| HCER.24_0 | -0.066391 | NA | 0 |
| HCER.24_1 | -0.0275562 | NA | 0 |
| LCER.14_0 | -0.0824827 | NA | 0 |
| LCER.16_0 | NA | 0.7604 | 0 |
| LCER.24_1 | NA | 0.7145 | 0 |
| LPC.15_0 | 0.10517546 | NA | 0 |
| LPC.17_0 | -0.1765654 | NA | 0 |
| LPC.20_4 | -0.0009497 | NA | 0 |
| LPC.22_6 | -0.0806304 | NA | 0 |
| LPE.16_0 | -0.007901 | NA | 0 |
| LPE.18_0 | -0.0296829 | NA | 0 |
| LPE.20_3 | -0.020689 | NA | 0 |
| LPE.22_6 | -0.0599751 | NA | 0 |
| MAG.18_2 | NA | 1.405 | 0 |
| MAG.18_3 | 0.01506807 | NA | 0 |
| PC.32_2 | -0.0175608 | NA | 0 |
| PC.34_1 | -0.0140936 | NA | 0 |
| PC.34_3 | -0.0106029 | NA | 0 |
| PC.35_2 | -0.0544087 | NA | 0 |
| PC.36_0 | 0.01328703 | NA | 0 |
| PC.36_4 | -0.0336402 | NA | 0 |
| PC.36_5 | -0.0292004 | NA | 0 |
| PC.38_2 | -0.0053732 | NA | 0 |
| PC.40_4 | 0.06036499 | NA | 0 |
| PC.40_5 | -0.0416406 | NA | 0 |
| PC.40_6 | 0.02766705 | NA | 0 |
| PC.40_7 | -0.0848486 | NA | 0 |
| PE.34_1 | NA | 1.35 | 0 |
| PE.34_2 | -0.0114346 | NA | 0 |
| PE.36_2 | NA | 1.283 | 0 |
| PE.38_3 | NA | 1.323 | 0 |
| PE.38_4 | NA | 1.273 | 0 |
| PE.40_4 | 0.05359989 | NA | 0 |
| PE_O.36_1 | 0.06109813 | NA | 0 |
| PE_O.36_4 | 0.14413805 | NA | 0 |
| PE_P.34_0 | -0.0670006 | NA | 0 |
| PE_P.34_2 | -0.0116205 | NA | 0 |
| PE_P.40_6 | -0.0243791 | NA | 0 |
| PE_P.40_7 | -0.0243624 | NA | 0 |
| PI.34_1 | 0.00860653 | NA | 0 |
| PI.36_2 | 0.01809374 | NA | 0 |
| PI.38_4 | 0.04089482 | NA | 0 |
| SM.14_0 | -0.1077039 | NA | 0 |
| SM.18_0 | 0.04449373 | NA | 0 |
| SM.18_1 | 0.03248881 | NA | 0 |
| SM.20_0 | 0.02069053 | NA | 0 |
| SM.20_1 | 0.03009467 | NA | 0 |
| SM.24_0 | 0.0031632 | NA | 0 |
| TAG.42_0 | -0.0730038 | NA | 0 |
| TAG.42_1 | NA | 1.289 | 0 |
| TAG.44_0 | NA | 1.365 | 0 |
| TAG.44_1 | NA | 1.431 | 0 |
| TAG.44_2 | NA | 1.412 | 0 |
| TAG.44_3 | NA | 1.442 | 0 |
| TAG.45_0 | NA | 1.35 | 0 |
| TAG.46_0 | NA | 1.436 | 0 |
| TAG.46_1 | NA | 1.543 | 0 |
| TAG.47_0 | NA | 1.497 | 0 |
| TAG.47_1 | NA | 1.601 | 0 |
| TAG.47_2 | NA | 1.545 | 0 |
| TAG.48_1 | NA | 1.655 | 0 |
| TAG.48_2 | NA | 1.633 | 0 |
| TAG.48_3 | NA | 1.661 | 0 |
| TAG.48_4 | NA | 1.666 | 0 |
| TAG.48_5 | NA | 1.41 | 0 |
| TAG.49_0 | NA | 1.594 | 0 |
| TAG.49_1 | NA | 1.729 | 0 |
| TAG.49_2 | NA | 1.749 | 0 |
| TAG.49_3 | NA | 1.755 | 0 |
| TAG.50_0 | NA | 1.621 | 0 |
| TAG.50_1 | NA | 1.656 | 0 |
| TAG.50_2 | NA | 1.687 | 0 |
| TAG.50_4 | NA | 1.747 | 0 |
| TAG.50_5 | NA | 1.692 | 0 |
| TAG.51_0 | NA | 1.663 | 0 |
| TAG.51_1 | NA | 1.682 | 0 |
| TAG.51_2 | NA | 1.766 | 0 |
| TAG.51_3 | NA | 1.854 | 0 |
| TAG.51_4 | NA | 1.767 | 0 |
| TAG.51_5 | NA | 1.562 | 0 |
| TAG.52_2 | NA | 1.748 | 0 |
| TAG.52_3 | NA | 1.75 | 0 |
| TAG.52_5 | NA | 1.739 | 0 |
| TAG.52_6 | NA | 1.656 | 0 |
| TAG.52_7 | NA | 1.604 | 0 |
| TAG.53_4 | NA | 1.678 | 0 |
| TAG.53_5 | NA | 1.834 | 0 |
| TAG.53_6 | NA | 1.746 | 0 |
| TAG.54_0 | NA | 1.479 | 0 |
| TAG.54_2 | NA | 1.659 | 0 |
| TAG.54_3 | NA | 1.697 | 0 |
| TAG.54_4 | NA | 1.708 | 0 |
| TAG.54_6 | NA | 1.691 | 0 |
| TAG.54_8 | NA | 1.456 | 0 |
| TAG.55_2 | NA | 1.493 | 0 |
| TAG.55_3 | NA | 1.578 | 0 |
| TAG.55_4 | NA | 1.603 | 0 |
| TAG.55_5 | NA | 1.628 | 0 |
| TAG.55_6 | NA | 1.722 | 0 |
| TAG.56_1 | -0.0336251 | NA | 0 |
| TAG.56_4 | NA | 1.57 | 0 |
| TAG.56_5 | NA | 1.577 | 0 |
| TAG.56_6 | NA | 1.607 | 0 |
| TAG.56_7 | NA | 1.597 | 0 |
| TAG.56_8 | NA | 1.539 | 0 |
| TAG.56_9 | NA | 1.339 | 0 |
| TAG.57_2 | NA | 1.334 | 0 |
| TAG.57_8 | NA | 1.389 | 0 |
| TAG.58_5 | NA | 1.558 | 0 |
| TAG.58_6 | NA | 1.534 | 0 |
| TAG.58_7 | NA | 1.577 | 0 |
| TAG.58_9 | NA | 1.343 | 0 |
| TAG.60_10 | -0.0604342 | NA | 0 |

^1^Lipid species are molecular lipid species grouped by total carbon and total double bond counts, log2 transformed and z-scaled

^2^Significant associations in individual regression are those with an FDR (BH) adjusted p-value < 0.05

^3^Interval censored (Weibull) Cox proportional hazards models adjusted for sociodemographics, health behaviors, and clinical variables

^4^Penalized (LASSO) regression model trained on a case-balanced 0.7 data subset with 10 fold cross-validation

Supplemental Figure 6. Volcano plot of associations between molecular lipid species and incident diabetes in individual interval censored (Weibull) Cox proportional hazards regression models adjusted for sociodemographics, health behaviors, and clinical variables. Hazard ratios are on the x-axis. Transformed (-log10) adjusted (FDR) p-values are on the y-axis. Top right quadrant = positively associated, top left quadrant = inversely associated, bottom left and right quadrants = insignificant. Red color with labels indicate molecular lipid species that were significant in individual regression and selected by the LASSO penalty in simultaneous penalized regression modeling. Penalized (LASSO) regression model trained on case-balanced 0.7 data with 10 fold cross-validation. Molecular lipid species are log2 transformed and z-scaled.

Supplemental table 6. Molecular lipid species^1^ significantly associated^2^ with incident diabetes in individual regression^3^ (n=526 lipids), penalized (LASSO) regression^4^ (n=96 lipids), or both (n=51 lipids).

| Molecular Lipid Species | LASSO Regression Beta Coefficient | Hazard ratio from individual regression models | Significant in both individual and LASSO regression models |
| --- | --- | --- | --- |
| DAG_12_0_18_1_uM | 0.03171076 | 1.605 | 1 |
| DAG_14_0_16_1_uM | 0.05983134 | 1.316 | 1 |
| DAG_16_0_18_2_uM | 0.00940594 | 1.776 | 1 |
| DAG_16_0_20_5_uM | 0.00572341 | 1.508 | 1 |
| DAG_16_1_16_1_uM | 0.1313448 | 1.712 | 1 |
| DAG_16_1_18_0_uM | 0.02826667 | 1.638 | 1 |
| DAG_16_1_20_2_uM | 0.107728 | 1.7 | 1 |
| DAG_18_1_20_4_uM | 0.0018718 | 1.452 | 1 |
| DAG_18_2_20_5_uM | -0.0243108 | 1.369 | 1 |
| DCER_22_0_uM | 0.03297978 | 1.348 | 1 |
| HCER_14_0_uM | -0.0531487 | 0.7729 | 1 |
| LCER_16_0_uM | -0.0205082 | 0.7604 | 1 |
| LCER_24_0_uM | -0.0536287 | 0.7331 | 1 |
| LCER_24_1_uM | -0.0540664 | 0.7145 | 1 |
| LPC_18_2_uM | -0.1025106 | 0.6869 | 1 |
| PE_18_1_18_1_uM | 0.01204297 | 1.279 | 1 |
| TAG44_2_FA12_0_uM | -0.1003659 | 1.301 | 1 |
| TAG44_2_FA16_0_uM | 0.03261894 | 1.467 | 1 |
| TAG44_2_FA18_2_uM | 0.00445492 | 1.378 | 1 |
| TAG46_3_FA16_1_uM | 0.04028668 | 1.735 | 1 |
| TAG46_3_FA18_1_uM | 0.00476608 | 1.456 | 1 |
| TAG46_3_FA18_2_uM | 0.02057842 | 1.564 | 1 |
| TAG47_2_FA18_2_uM | -0.0645777 | 1.592 | 1 |
| TAG50_1_FA16_0_uM | 0.0184868 | 1.735 | 1 |
| TAG50_3_FA14_1_uM | -0.0754885 | 1.607 | 1 |
| TAG50_3_FA16_1_uM | 0.00482105 | 1.823 | 1 |
| TAG50_4_FA18_1_uM | -0.0187741 | 1.7 | 1 |
| TAG50_4_FA20_4_uM | 0.0080011 | 1.497 | 1 |
| TAG50_5_FA14_1_uM | 0.07068659 | 1.669 | 1 |
| TAG51_3_FA16_0_uM | 0.05928106 | 2.491 | 1 |
| TAG51_4_FA16_0_uM | 0.02578043 | 2.382 | 1 |
| TAG52_1_FA16_0_uM | 0.00869513 | 1.658 | 1 |
| TAG52_4_FA16_0_uM | 0.03802846 | 1.655 | 1 |
| TAG52_4_FA20_0_uM | 0.00350406 | 1.614 | 1 |
| TAG52_6_FA20_5_uM | -0.0365151 | 1.493 | 1 |
| TAG52_7_FA16_0_uM | 0.0032717 | 1.577 | 1 |
| TAG52_8_FA18_2_uM | 0.06468013 | 1.985 | 1 |
| TAG53_5_FA18_1_uM | 0.05307549 | 1.515 | 1 |
| TAG54_0_FA18_0_uM | 0.1035002 | 1.466 | 1 |
| TAG54_1_FA20_1_uM | 0.04860102 | 1.664 | 1 |
| TAG54_2_FA18_0_uM | 0.09314143 | 1.57 | 1 |
| TAG54_5_FA20_5_uM | -0.0511383 | 1.387 | 1 |
| TAG54_8_FA22_6_uM | -0.0300352 | 1.543 | 1 |
| TAG55_2_FA18_1_uM | -0.0021863 | 1.49 | 1 |
| TAG55_5_FA18_2_uM | 0.00016043 | 1.522 | 1 |
| TAG55_6_FA18_1_uM | -0.0357928 | 1.608 | 1 |
| TAG56_2_FA20_0_uM | 0.02548378 | 1.392 | 1 |
| TAG56_3_FA18_2_uM | 0.01531837 | 1.44 | 1 |
| TAG56_8_FA16_0_uM | 0.06765545 | 1.628 | 1 |
| TAG58_7_FA22_5_uM | -0.0087848 | 1.464 | 1 |
| TAG58_9_FA22_6_uM | -0.0344695 | 1.29 | 1 |
| CE_14_0_uM | NA | 1.268 | 0 |
| CE_14_1_uM | NA | 1.303 | 0 |
| CE_20_2_uM | 0.06794753 | NA | 0 |
| CE_20_3_uM | 0.1119756 | NA | 0 |
| CE_22_1_uM | -0.0128614 | NA | 0 |
| CE_22_2_uM | 0.04665169 | NA | 0 |
| CER_14_0_uM | -0.0944466 | NA | 0 |
| CER_18_0_uM | NA | 1.404 | 0 |
| CER_18_1_uM | 0.02904761 | NA | 0 |
| CER_20_1_uM | 0.1025221 | NA | 0 |
| CER_22_1_uM | NA | 1.335 | 0 |
| CER_24_0_uM | -0.0152014 | NA | 0 |
| CER_26_0_uM | -0.0142071 | NA | 0 |
| DAG_14_0_18_1_uM | NA | 1.776 | 0 |
| DAG_14_0_18_2_uM | NA | 1.736 | 0 |
| DAG_14_0_20_4_uM | NA | 1.642 | 0 |
| DAG_14_1_16_0_uM | 0.03326606 | NA | 0 |
| DAG_14_1_18_1_uM | NA | 1.537 | 0 |
| DAG_15_0_18_1_uM | NA | 1.653 | 0 |
| DAG_15_0_18_2_uM | 0.00288252 | NA | 0 |
| DAG_16_0_16_0_uM | 0.05155497 | NA | 0 |
| DAG_16_0_16_1_uM | NA | 1.726 | 0 |
| DAG_16_0_18_0_uM | NA | 1.321 | 0 |
| DAG_16_0_18_1_uM | NA | 1.706 | 0 |
| DAG_16_0_18_3_uM | NA | 1.678 | 0 |
| DAG_16_0_20_3_uM | NA | 1.478 | 0 |
| DAG_16_0_20_4_uM | NA | 1.484 | 0 |
| DAG_16_0_22_5_uM | NA | 1.715 | 0 |
| DAG_16_0_22_6_uM | NA | 1.58 | 0 |
| DAG_16_1_18_1_uM | NA | 1.697 | 0 |
| DAG_16_1_18_2_uM | NA | 1.784 | 0 |
| DAG_16_1_18_3_uM | NA | 1.643 | 0 |
| DAG_16_1_20_4_uM | NA | 1.454 | 0 |
| DAG_16_1_22_6_uM | NA | 1.411 | 0 |
| DAG_18_0_18_1_uM | NA | 1.623 | 0 |
| DAG_18_0_18_2_uM | NA | 1.714 | 0 |
| DAG_18_0_18_3_uM | NA | 1.6 | 0 |
| DAG_18_1_18_1_uM | NA | 1.63 | 0 |
| DAG_18_1_18_2_uM | NA | 1.662 | 0 |
| DAG_18_1_20_1_uM | NA | 1.533 | 0 |
| DAG_18_1_20_2_uM | NA | 1.417 | 0 |
| DAG_18_1_20_3_uM | NA | 1.409 | 0 |
| DAG_18_1_20_5_uM | NA | 1.335 | 0 |
| DAG_18_1_22_4_uM | NA | 1.526 | 0 |
| DAG_18_1_22_5_uM | NA | 1.519 | 0 |
| DAG_18_1_22_6_uM | NA | 1.448 | 0 |
| DAG_18_2_18_3_uM | NA | 1.486 | 0 |
| DAG_18_2_20_3_uM | NA | 1.451 | 0 |
| DAG_18_2_20_4_uM | NA | 1.483 | 0 |
| DAG_18_2_22_4_uM | NA | 1.455 | 0 |
| DAG_18_2_22_5_uM | NA | 1.542 | 0 |
| DAG_18_2_22_6_uM | NA | 1.401 | 0 |
| DCER_18_0_uM | 0.0219722 | NA | 0 |
| DCER_22_2_uM | NA | 1.381 | 0 |
| HCER_24_0_uM | -0.0497326 | NA | 0 |
| HCER_24_1_uM | -0.016069 | NA | 0 |
| LCER_18_0_uM | NA | 0.7634 | 0 |
| LCER_22_0_uM | -0.0034485 | NA | 0 |
| LPC_15_0_uM | 0.04380619 | NA | 0 |
| LPC_17_0_uM | -0.0552375 | NA | 0 |
| LPC_18_1_uM | NA | 0.7423 | 0 |
| LPC_22_6_uM | -0.0968441 | NA | 0 |
| LPE_20_3_uM | -0.0204465 | NA | 0 |
| LPE_22_5_uM | 0.02818934 | NA | 0 |
| LPE_22_6_uM | -0.067702 | NA | 0 |
| MAG_16_1_uM | 0.00059058 | NA | 0 |
| MAG_18_2_uM | NA | 1.405 | 0 |
| PC_14_0_18_2_uM | -0.0189633 | NA | 0 |
| PC_16_0_16_0_uM | -0.0700549 | NA | 0 |
| PC_16_0_20_3_uM | NA | 1.287 | 0 |
| PC_16_0_20_4_uM | -0.0176361 | NA | 0 |
| PC_17_0_18_2_uM | -0.1067434 | NA | 0 |
| PC_18_0_20_3_uM | NA | 1.301 | 0 |
| PC_18_0_20_4_uM | 0.1250478 | NA | 0 |
| PC_18_1_20_2_uM | 0.01781172 | NA | 0 |
| PC_18_1_20_5_uM | 0.01135253 | NA | 0 |
| PC_18_2_16_1_uM | -0.0020563 | NA | 0 |
| PC_18_2_20_3_uM | -0.0049182 | NA | 0 |
| PC_18_2_20_4_uM | -0.0865201 | NA | 0 |
| PE_16_0_18_1_uM | NA | 1.35 | 0 |
| PE_16_0_22_5_uM | NA | 1.331 | 0 |
| PE_18_0_20_3_uM | NA | 1.323 | 0 |
| PE_18_0_20_4_uM | NA | 1.273 | 0 |
| PE_18_0_22_5_uM | NA | 1.335 | 0 |
| PE_O_16_0_20_4_uM | 0.02441963 | NA | 0 |
| PE_O_18_0_18_1_uM | 0.00658995 | NA | 0 |
| PE_P_16_0_22_6_uM | -0.0169009 | NA | 0 |
| PE_P_18_0_22_6_uM | -0.0616329 | NA | 0 |
| PI_18_0_20_4_uM | 0.03793503 | NA | 0 |
| SM_18_0_uM | 0.00874048 | NA | 0 |
| SM_18_1_uM | 0.09331981 | NA | 0 |
| TAG42_0_FA16_0_uM | NA | 1.359 | 0 |
| TAG42_1_FA16_0_uM | NA | 1.392 | 0 |
| TAG42_1_FA18_1_uM | -0.013491 | NA | 0 |
| TAG44_0_FA14_0_uM | NA | 1.449 | 0 |
| TAG44_0_FA16_0_uM | NA | 1.392 | 0 |
| TAG44_0_FA18_0_uM | NA | 1.278 | 0 |
| TAG44_1_FA12_0_uM | NA | 1.386 | 0 |
| TAG44_1_FA14_0_uM | NA | 1.568 | 0 |
| TAG44_1_FA16_0_uM | NA | 1.454 | 0 |
| TAG44_1_FA18_1_uM | NA | 1.371 | 0 |
| TAG44_2_FA16_1_uM | NA | 1.438 | 0 |
| TAG44_2_FA18_1_uM | NA | 1.464 | 0 |
| TAG44_3_FA18_2_uM | NA | 1.442 | 0 |
| TAG45_0_FA16_0_uM | NA | 1.35 | 0 |
| TAG46_0_FA14_0_uM | NA | 1.562 | 0 |
| TAG46_0_FA16_0_uM | NA | 1.512 | 0 |
| TAG46_1_FA12_0_uM | NA | 1.492 | 0 |
| TAG46_1_FA14_0_uM | NA | 1.589 | 0 |
| TAG46_1_FA14_1_uM | NA | 1.501 | 0 |
| TAG46_1_FA16_0_uM | NA | 1.54 | 0 |
| TAG46_1_FA16_1_uM | NA | 1.655 | 0 |
| TAG46_1_FA18_0_uM | NA | 1.322 | 0 |
| TAG46_1_FA18_1_uM | NA | 1.498 | 0 |
| TAG46_2_FA12_0_uM | NA | 1.566 | 0 |
| TAG46_2_FA14_0_uM | NA | 1.643 | 0 |
| TAG46_2_FA14_1_uM | NA | 1.611 | 0 |
| TAG46_2_FA16_0_uM | NA | 1.542 | 0 |
| TAG46_2_FA16_1_uM | NA | 1.609 | 0 |
| TAG46_2_FA18_1_uM | NA | 1.497 | 0 |
| TAG46_2_FA18_2_uM | NA | 1.535 | 0 |
| TAG46_3_FA12_0_uM | NA | 1.482 | 0 |
| TAG46_3_FA14_0_uM | NA | 1.581 | 0 |
| TAG46_3_FA14_1_uM | NA | 1.416 | 0 |
| TAG46_3_FA16_0_uM | NA | 1.614 | 0 |
| TAG46_3_FA18_3_uM | NA | 1.364 | 0 |
| TAG46_4_FA18_2_uM | NA | 1.492 | 0 |
| TAG47_0_FA15_0_uM | NA | 1.435 | 0 |
| TAG47_0_FA16_0_uM | NA | 1.514 | 0 |
| TAG47_0_FA17_0_uM | NA | 1.45 | 0 |
| TAG47_1_FA14_0_uM | NA | 1.525 | 0 |
| TAG47_1_FA15_0_uM | NA | 1.537 | 0 |
| TAG47_1_FA16_0_uM | NA | 1.642 | 0 |
| TAG47_1_FA16_1_uM | NA | 1.492 | 0 |
| TAG47_1_FA17_0_uM | NA | 1.276 | 0 |
| TAG47_1_FA18_1_uM | NA | 1.57 | 0 |
| TAG47_2_FA14_0_uM | NA | 1.436 | 0 |
| TAG47_2_FA15_0_uM | NA | 1.434 | 0 |
| TAG47_2_FA18_1_uM | NA | 1.464 | 0 |
| TAG48_0_FA14_0_uM | NA | 1.569 | 0 |
| TAG48_0_FA16_0_uM | NA | 1.593 | 0 |
| TAG48_0_FA18_0_uM | NA | 1.585 | 0 |
| TAG48_1_FA12_0_uM | NA | 1.51 | 0 |
| TAG48_1_FA14_0_uM | NA | 1.64 | 0 |
| TAG48_1_FA14_1_uM | NA | 1.531 | 0 |
| TAG48_1_FA16_0_uM | NA | 1.629 | 0 |
| TAG48_1_FA16_1_uM | NA | 1.669 | 0 |
| TAG48_1_FA18_0_uM | NA | 1.559 | 0 |
| TAG48_1_FA18_1_uM | NA | 1.646 | 0 |
| TAG48_2_FA12_0_uM | NA | 1.443 | 0 |
| TAG48_2_FA14_0_uM | NA | 1.719 | 0 |
| TAG48_2_FA14_1_uM | NA | 1.63 | 0 |
| TAG48_2_FA16_0_uM | NA | 1.702 | 0 |
| TAG48_2_FA16_1_uM | NA | 1.676 | 0 |
| TAG48_2_FA18_0_uM | NA | 1.336 | 0 |
| TAG48_2_FA18_1_uM | NA | 1.588 | 0 |
| TAG48_2_FA18_2_uM | NA | 1.695 | 0 |
| TAG48_3_FA12_0_uM | NA | 1.447 | 0 |
| TAG48_3_FA14_0_uM | NA | 1.721 | 0 |
| TAG48_3_FA14_1_uM | NA | 1.716 | 0 |
| TAG48_3_FA16_0_uM | NA | 1.653 | 0 |
| TAG48_3_FA16_1_uM | NA | 1.76 | 0 |
| TAG48_3_FA18_1_uM | NA | 1.538 | 0 |
| TAG48_3_FA18_2_uM | NA | 1.617 | 0 |
| TAG48_3_FA18_3_uM | NA | 1.592 | 0 |
| TAG48_4_FA12_0_uM | NA | 1.398 | 0 |
| TAG48_4_FA14_0_uM | NA | 1.66 | 0 |
| TAG48_4_FA14_1_uM | NA | 1.777 | 0 |
| TAG48_4_FA16_0_uM | NA | 1.743 | 0 |
| TAG48_4_FA16_1_uM | NA | 1.973 | 0 |
| TAG48_4_FA18_1_uM | NA | 1.605 | 0 |
| TAG48_4_FA18_2_uM | NA | 1.489 | 0 |
| TAG48_4_FA18_3_uM | NA | 1.578 | 0 |
| TAG48_5_FA18_2_uM | NA | 1.492 | 0 |
| TAG48_5_FA18_3_uM | NA | 1.307 | 0 |
| TAG49_0_FA15_0_uM | NA | 1.675 | 0 |
| TAG49_0_FA16_0_uM | NA | 1.606 | 0 |
| TAG49_0_FA17_0_uM | NA | 1.698 | 0 |
| TAG49_0_FA18_0_uM | NA | 1.404 | 0 |
| TAG49_1_FA14_0_uM | NA | 1.785 | 0 |
| TAG49_1_FA15_0_uM | NA | 1.678 | 0 |
| TAG49_1_FA16_0_uM | NA | 1.69 | 0 |
| TAG49_1_FA16_1_uM | NA | 1.691 | 0 |
| TAG49_1_FA17_0_uM | NA | 1.771 | 0 |
| TAG49_1_FA18_1_uM | NA | 1.685 | 0 |
| TAG49_2_FA14_0_uM | NA | 1.812 | 0 |
| TAG49_2_FA15_0_uM | NA | 1.763 | 0 |
| TAG49_2_FA16_0_uM | NA | 1.768 | 0 |
| TAG49_2_FA16_1_uM | NA | 1.737 | 0 |
| TAG49_2_FA17_0_uM | NA | 1.623 | 0 |
| TAG49_2_FA18_1_uM | NA | 1.636 | 0 |
| TAG49_2_FA18_2_uM | NA | 1.749 | 0 |
| TAG49_3_FA15_0_uM | NA | 1.805 | 0 |
| TAG49_3_FA16_0_uM | NA | 1.793 | 0 |
| TAG49_3_FA16_1_uM | NA | 1.794 | 0 |
| TAG49_3_FA18_2_uM | NA | 1.813 | 0 |
| TAG49_3_FA18_3_uM | NA | 1.477 | 0 |
| TAG50_0_FA14_0_uM | NA | 1.523 | 0 |
| TAG50_0_FA16_0_uM | NA | 1.628 | 0 |
| TAG50_0_FA18_0_uM | NA | 1.579 | 0 |
| TAG50_1_FA14_0_uM | NA | 1.624 | 0 |
| TAG50_1_FA16_1_uM | NA | 1.718 | 0 |
| TAG50_1_FA18_0_uM | NA | 1.616 | 0 |
| TAG50_1_FA18_1_uM | NA | 1.723 | 0 |
| TAG50_1_FA20_1_uM | NA | 1.368 | 0 |
| TAG50_2_FA14_0_uM | NA | 1.658 | 0 |
| TAG50_2_FA14_1_uM | NA | 1.553 | 0 |
| TAG50_2_FA16_0_uM | NA | 1.822 | 0 |
| TAG50_2_FA16_1_uM | NA | 1.753 | 0 |
| TAG50_2_FA18_0_uM | NA | 1.679 | 0 |
| TAG50_2_FA18_1_uM | NA | 1.711 | 0 |
| TAG50_2_FA18_2_uM | NA | 1.803 | 0 |
| TAG50_2_FA20_2_uM | NA | 1.315 | 0 |
| TAG50_3_FA14_0_uM | NA | 1.727 | 0 |
| TAG50_3_FA16_0_uM | NA | 1.814 | 0 |
| TAG50_3_FA18_0_uM | NA | 1.75 | 0 |
| TAG50_3_FA18_1_uM | NA | 1.755 | 0 |
| TAG50_3_FA18_2_uM | NA | 1.771 | 0 |
| TAG50_3_FA18_3_uM | NA | 1.639 | 0 |
| TAG50_3_FA20_3_uM | NA | 1.47 | 0 |
| TAG50_4_FA14_0_uM | NA | 1.656 | 0 |
| TAG50_4_FA14_1_uM | NA | 1.615 | 0 |
| TAG50_4_FA16_0_uM | NA | 1.763 | 0 |
| TAG50_4_FA16_1_uM | NA | 1.794 | 0 |
| TAG50_4_FA18_2_uM | NA | 1.71 | 0 |
| TAG50_4_FA18_3_uM | NA | 1.662 | 0 |
| TAG50_5_FA14_0_uM | NA | 1.723 | 0 |
| TAG50_5_FA16_0_uM | NA | 1.55 | 0 |
| TAG50_5_FA16_1_uM | NA | 1.818 | 0 |
| TAG50_5_FA18_1_uM | NA | 1.687 | 0 |
| TAG50_5_FA18_2_uM | NA | 1.68 | 0 |
| TAG50_5_FA18_3_uM | NA | 1.643 | 0 |
| TAG51_0_FA16_0_uM | NA | 1.586 | 0 |
| TAG51_0_FA17_0_uM | NA | 1.864 | 0 |
| TAG51_0_FA18_0_uM | NA | 1.599 | 0 |
| TAG51_1_FA15_0_uM | NA | 1.54 | 0 |
| TAG51_1_FA16_0_uM | NA | 1.722 | 0 |
| TAG51_1_FA17_0_uM | NA | 1.711 | 0 |
| TAG51_1_FA18_0_uM | NA | 1.662 | 0 |
| TAG51_1_FA18_1_uM | NA | 1.725 | 0 |
| TAG51_2_FA15_0_uM | NA | 1.647 | 0 |
| TAG51_2_FA16_0_uM | NA | 1.835 | 0 |
| TAG51_2_FA16_1_uM | NA | 1.726 | 0 |
| TAG51_2_FA17_0_uM | NA | 1.839 | 0 |
| TAG51_2_FA18_0_uM | NA | 1.682 | 0 |
| TAG51_2_FA18_1_uM | NA | 1.704 | 0 |
| TAG51_2_FA18_2_uM | NA | 1.763 | 0 |
| TAG51_3_FA15_0_uM | NA | 1.638 | 0 |
| TAG51_3_FA16_1_uM | NA | 1.802 | 0 |
| TAG51_3_FA17_0_uM | NA | 1.813 | 0 |
| TAG51_3_FA18_1_uM | NA | 1.82 | 0 |
| TAG51_3_FA18_2_uM | NA | 1.756 | 0 |
| TAG51_3_FA18_3_uM | NA | 1.714 | 0 |
| TAG51_4_FA15_0_uM | NA | 1.595 | 0 |
| TAG51_4_FA16_1_uM | NA | 1.997 | 0 |
| TAG51_4_FA18_1_uM | NA | 1.761 | 0 |
| TAG51_4_FA18_2_uM | NA | 1.659 | 0 |
| TAG51_4_FA18_3_uM | NA | 1.643 | 0 |
| TAG51_5_FA18_2_uM | NA | 1.525 | 0 |
| TAG51_5_FA18_3_uM | NA | 1.59 | 0 |
| TAG52_0_FA16_0_uM | NA | 1.59 | 0 |
| TAG52_0_FA18_0_uM | NA | 1.565 | 0 |
| TAG52_0_FA20_0_uM | NA | 1.648 | 0 |
| TAG52_1_FA16_1_uM | NA | 1.669 | 0 |
| TAG52_1_FA18_0_uM | NA | 1.632 | 0 |
| TAG52_1_FA18_1_uM | NA | 1.643 | 0 |
| TAG52_1_FA20_0_uM | NA | 1.641 | 0 |
| TAG52_1_FA20_1_uM | NA | 1.723 | 0 |
| TAG52_2_FA14_0_uM | NA | 1.508 | 0 |
| TAG52_2_FA16_0_uM | NA | 1.71 | 0 |
| TAG52_2_FA16_1_uM | NA | 1.679 | 0 |
| TAG52_2_FA18_0_uM | NA | 1.726 | 0 |
| TAG52_2_FA18_1_uM | NA | 1.71 | 0 |
| TAG52_2_FA18_2_uM | NA | 1.775 | 0 |
| TAG52_2_FA20_0_uM | NA | 1.751 | 0 |
| TAG52_2_FA20_1_uM | NA | 1.717 | 0 |
| TAG52_2_FA20_2_uM | NA | 1.71 | 0 |
| TAG52_3_FA14_0_uM | NA | 1.716 | 0 |
| TAG52_3_FA16_0_uM | NA | 1.759 | 0 |
| TAG52_3_FA16_1_uM | NA | 1.65 | 0 |
| TAG52_3_FA18_0_uM | NA | 1.771 | 0 |
| TAG52_3_FA18_1_uM | NA | 1.776 | 0 |
| TAG52_3_FA18_2_uM | NA | 1.742 | 0 |
| TAG52_3_FA18_3_uM | NA | 1.686 | 0 |
| TAG52_3_FA20_0_uM | NA | 1.731 | 0 |
| TAG52_3_FA20_1_uM | NA | 1.757 | 0 |
| TAG52_3_FA20_2_uM | NA | 1.684 | 0 |
| TAG52_3_FA20_3_uM | NA | 1.635 | 0 |
| TAG52_3_FA22_1_uM | NA | 1.419 | 0 |
| TAG52_4_FA14_0_uM | NA | 1.717 | 0 |
| TAG52_4_FA16_1_uM | NA | 1.654 | 0 |
| TAG52_4_FA18_0_uM | NA | 1.738 | 0 |
| TAG52_4_FA18_1_uM | NA | 1.712 | 0 |
| TAG52_4_FA18_2_uM | NA | 1.648 | 0 |
| TAG52_4_FA18_3_uM | NA | 1.702 | 0 |
| TAG52_4_FA20_2_uM | NA | 1.763 | 0 |
| TAG52_4_FA20_3_uM | NA | 1.627 | 0 |
| TAG52_4_FA20_4_uM | NA | 1.549 | 0 |
| TAG52_4_FA22_1_uM | NA | 1.889 | 0 |
| TAG52_4_FA22_4_uM | NA | 1.317 | 0 |
| TAG52_5_FA14_0_uM | NA | 1.618 | 0 |
| TAG52_5_FA16_0_uM | NA | 1.678 | 0 |
| TAG52_5_FA16_1_uM | NA | 1.574 | 0 |
| TAG52_5_FA18_1_uM | NA | 1.651 | 0 |
| TAG52_5_FA18_2_uM | NA | 1.612 | 0 |
| TAG52_5_FA18_3_uM | NA | 1.653 | 0 |
| TAG52_5_FA20_3_uM | NA | 1.696 | 0 |
| TAG52_5_FA20_4_uM | NA | 1.537 | 0 |
| TAG52_5_FA20_5_uM | NA | 1.605 | 0 |
| TAG52_5_FA22_5_uM | NA | 1.746 | 0 |
| TAG52_6_FA14_0_uM | NA | 1.644 | 0 |
| TAG52_6_FA16_0_uM | NA | 1.575 | 0 |
| TAG52_6_FA16_1_uM | NA | 1.608 | 0 |
| TAG52_6_FA18_1_uM | NA | 1.568 | 0 |
| TAG52_6_FA18_2_uM | NA | 1.538 | 0 |
| TAG52_6_FA18_3_uM | NA | 1.551 | 0 |
| TAG52_6_FA20_4_uM | NA | 1.513 | 0 |
| TAG52_6_FA22_6_uM | NA | 1.57 | 0 |
| TAG52_7_FA18_1_uM | NA | 1.946 | 0 |
| TAG52_7_FA20_5_uM | NA | 1.345 | 0 |
| TAG52_7_FA22_6_uM | NA | 1.383 | 0 |
| TAG52_8_FA16_1_uM | NA | 1.567 | 0 |
| TAG53_0_FA16_0_uM | NA | 1.596 | 0 |
| TAG53_1_FA16_0_uM | NA | 1.598 | 0 |
| TAG53_1_FA17_0_uM | NA | 1.635 | 0 |
| TAG53_1_FA18_0_uM | NA | 1.616 | 0 |
| TAG53_1_FA18_1_uM | NA | 1.598 | 0 |
| TAG53_2_FA16_0_uM | NA | 1.735 | 0 |
| TAG53_2_FA17_0_uM | NA | 1.629 | 0 |
| TAG53_2_FA18_0_uM | NA | 1.69 | 0 |
| TAG53_2_FA18_1_uM | NA | 1.61 | 0 |
| TAG53_2_FA18_2_uM | NA | 1.679 | 0 |
| TAG53_3_FA16_0_uM | NA | 1.789 | 0 |
| TAG53_3_FA17_0_uM | NA | 1.643 | 0 |
| TAG53_3_FA18_1_uM | NA | 1.667 | 0 |
| TAG53_3_FA18_2_uM | NA | 1.669 | 0 |
| TAG53_4_FA16_0_uM | NA | 1.816 | 0 |
| TAG53_4_FA17_0_uM | NA | 1.522 | 0 |
| TAG53_4_FA18_1_uM | NA | 1.567 | 0 |
| TAG53_4_FA18_2_uM | NA | 1.615 | 0 |
| TAG53_4_FA18_3_uM | NA | 1.71 | 0 |
| TAG53_4_FA20_4_uM | NA | 1.538 | 0 |
| TAG53_5_FA18_2_uM | NA | 2.013 | 0 |
| TAG53_5_FA18_3_uM | NA | 1.622 | 0 |
| TAG53_5_FA20_4_uM | NA | 1.507 | 0 |
| TAG53_6_FA18_1_uM | NA | 1.557 | 0 |
| TAG53_6_FA18_2_uM | NA | 1.578 | 0 |
| TAG53_6_FA18_3_uM | NA | 1.475 | 0 |
| TAG53_6_FA20_4_uM | NA | 1.391 | 0 |
| TAG53_7_FA18_3_uM | NA | 1.511 | 0 |
| TAG54_0_FA16_0_uM | NA | 1.416 | 0 |
| TAG54_1_FA16_0_uM | NA | 1.504 | 0 |
| TAG54_1_FA18_0_uM | NA | 1.508 | 0 |
| TAG54_1_FA18_1_uM | NA | 1.518 | 0 |
| TAG54_1_FA20_0_uM | NA | 1.58 | 0 |
| TAG54_2_FA16_0_uM | NA | 1.658 | 0 |
| TAG54_2_FA18_1_uM | NA | 1.556 | 0 |
| TAG54_2_FA18_2_uM | NA | 1.61 | 0 |
| TAG54_2_FA20_0_uM | NA | 1.645 | 0 |
| TAG54_2_FA20_1_uM | NA | 1.704 | 0 |
| TAG54_2_FA20_2_uM | NA | 1.581 | 0 |
| TAG54_3_FA16_0_uM | NA | 1.72 | 0 |
| TAG54_3_FA16_1_uM | NA | 1.634 | 0 |
| TAG54_3_FA18_0_uM | NA | 1.622 | 0 |
| TAG54_3_FA18_1_uM | NA | 1.568 | 0 |
| TAG54_3_FA18_2_uM | NA | 1.613 | 0 |
| TAG54_3_FA18_3_uM | NA | 1.667 | 0 |
| TAG54_3_FA20_1_uM | NA | 1.783 | 0 |
| TAG54_3_FA20_2_uM | NA | 1.654 | 0 |
| TAG54_3_FA20_3_uM | NA | 1.622 | 0 |
| TAG54_4_FA16_0_uM | NA | 1.733 | 0 |
| TAG54_4_FA16_1_uM | NA | 1.661 | 0 |
| TAG54_4_FA18_0_uM | NA | 1.574 | 0 |
| TAG54_4_FA18_1_uM | NA | 1.539 | 0 |
| TAG54_4_FA18_2_uM | NA | 1.558 | 0 |
| TAG54_4_FA18_3_uM | NA | 1.569 | 0 |
| TAG54_4_FA20_1_uM | NA | 1.732 | 0 |
| TAG54_4_FA20_2_uM | NA | 1.71 | 0 |
| TAG54_4_FA20_3_uM | NA | 1.66 | 0 |
| TAG54_4_FA20_4_uM | NA | 1.553 | 0 |
| TAG54_4_FA22_1_uM | NA | 1.654 | 0 |
| TAG54_4_FA22_4_uM | NA | 1.682 | 0 |
| TAG54_5_FA16_0_uM | NA | 1.707 | 0 |
| TAG54_5_FA16_1_uM | NA | 1.739 | 0 |
| TAG54_5_FA18_0_uM | NA | 1.592 | 0 |
| TAG54_5_FA18_1_uM | NA | 1.5 | 0 |
| TAG54_5_FA18_2_uM | NA | 1.49 | 0 |
| TAG54_5_FA18_3_uM | NA | 1.52 | 0 |
| TAG54_5_FA20_2_uM | NA | 1.697 | 0 |
| TAG54_5_FA20_3_uM | NA | 1.705 | 0 |
| TAG54_5_FA20_4_uM | NA | 1.531 | 0 |
| TAG54_5_FA22_1_uM | NA | 1.462 | 0 |
| TAG54_5_FA22_4_uM | NA | 1.679 | 0 |
| TAG54_5_FA22_5_uM | NA | 1.744 | 0 |
| TAG54_6_FA16_0_uM | NA | 1.677 | 0 |
| TAG54_6_FA16_1_uM | NA | 1.652 | 0 |
| TAG54_6_FA18_1_uM | NA | 1.481 | 0 |
| TAG54_6_FA18_2_uM | NA | 1.448 | 0 |
| TAG54_6_FA18_3_uM | NA | 1.428 | 0 |
| TAG54_6_FA20_3_uM | NA | 1.629 | 0 |
| TAG54_6_FA20_4_uM | NA | 1.609 | 0 |
| TAG54_6_FA20_5_uM | NA | 1.435 | 0 |
| TAG54_6_FA22_5_uM | NA | 1.686 | 0 |
| TAG54_6_FA22_6_uM | NA | 1.62 | 0 |
| TAG54_7_FA16_1_uM | NA | 1.626 | 0 |
| TAG54_7_FA18_1_uM | NA | 1.438 | 0 |
| TAG54_7_FA18_2_uM | NA | 1.389 | 0 |
| TAG54_7_FA18_3_uM | NA | 1.369 | 0 |
| TAG54_7_FA20_4_uM | NA | 1.554 | 0 |
| TAG54_7_FA20_5_uM | NA | 1.482 | 0 |
| TAG54_7_FA22_5_uM | NA | 1.695 | 0 |
| TAG54_7_FA22_6_uM | NA | 1.564 | 0 |
| TAG54_8_FA18_2_uM | NA | 1.356 | 0 |
| TAG54_8_FA18_3_uM | NA | 1.305 | 0 |
| TAG54_8_FA20_4_uM | NA | 1.322 | 0 |
| TAG54_8_FA20_5_uM | NA | 1.399 | 0 |
| TAG55_1_FA16_0_uM | NA | 1.613 | 0 |
| TAG55_1_FA18_1_uM | NA | 1.436 | 0 |
| TAG55_2_FA18_2_uM | NA | 1.328 | 0 |
| TAG55_3_FA18_1_uM | NA | 1.588 | 0 |
| TAG55_3_FA18_2_uM | NA | 1.57 | 0 |
| TAG55_4_FA18_1_uM | NA | 1.608 | 0 |
| TAG55_4_FA18_2_uM | NA | 1.58 | 0 |
| TAG55_5_FA18_1_uM | NA | 1.594 | 0 |
| TAG55_5_FA20_4_uM | NA | 1.565 | 0 |
| TAG55_6_FA20_3_uM | NA | 1.71 | 0 |
| TAG55_6_FA20_4_uM | NA | 1.483 | 0 |
| TAG55_7_FA18_1_uM | NA | 1.483 | 0 |
| TAG55_7_FA22_6_uM | NA | 1.511 | 0 |
| TAG56_10_FA18_2_uM | NA | 1.611 | 0 |
| TAG56_2_FA16_0_uM | -0.0094859 | NA | 0 |
| TAG56_2_FA18_0_uM | NA | 1.578 | 0 |
| TAG56_2_FA18_1_uM | NA | 1.35 | 0 |
| TAG56_2_FA20_1_uM | NA | 1.521 | 0 |
| TAG56_3_FA16_0_uM | NA | 1.469 | 0 |
| TAG56_3_FA18_0_uM | NA | 1.544 | 0 |
| TAG56_3_FA18_1_uM | NA | 1.499 | 0 |
| TAG56_3_FA20_0_uM | NA | 1.443 | 0 |
| TAG56_3_FA20_1_uM | NA | 1.482 | 0 |
| TAG56_3_FA20_2_uM | NA | 1.59 | 0 |
| TAG56_4_FA16_0_uM | NA | 1.608 | 0 |
| TAG56_4_FA18_0_uM | NA | 1.575 | 0 |
| TAG56_4_FA18_1_uM | NA | 1.562 | 0 |
| TAG56_4_FA18_2_uM | NA | 1.484 | 0 |
| TAG56_4_FA20_1_uM | NA | 1.414 | 0 |
| TAG56_4_FA20_2_uM | NA | 1.489 | 0 |
| TAG56_4_FA20_3_uM | NA | 1.531 | 0 |
| TAG56_4_FA20_4_uM | NA | 1.48 | 0 |
| TAG56_4_FA22_4_uM | NA | 1.644 | 0 |
| TAG56_5_FA16_0_uM | NA | 1.637 | 0 |
| TAG56_5_FA18_0_uM | NA | 1.525 | 0 |
| TAG56_5_FA18_1_uM | NA | 1.577 | 0 |
| TAG56_5_FA18_2_uM | NA | 1.5 | 0 |
| TAG56_5_FA20_1_uM | NA | 1.381 | 0 |
| TAG56_5_FA20_2_uM | NA | 1.422 | 0 |
| TAG56_5_FA20_3_uM | NA | 1.47 | 0 |
| TAG56_5_FA20_4_uM | NA | 1.497 | 0 |
| TAG56_5_FA22_4_uM | NA | 1.621 | 0 |
| TAG56_5_FA22_5_uM | NA | 1.602 | 0 |
| TAG56_6_FA16_0_uM | NA | 1.662 | 0 |
| TAG56_6_FA18_0_uM | NA | 1.499 | 0 |
| TAG56_6_FA18_1_uM | NA | 1.555 | 0 |
| TAG56_6_FA18_2_uM | NA | 1.583 | 0 |
| TAG56_6_FA18_3_uM | NA | 1.655 | 0 |
| TAG56_6_FA20_2_uM | NA | 1.375 | 0 |
| TAG56_6_FA20_3_uM | NA | 1.404 | 0 |
| TAG56_6_FA20_4_uM | NA | 1.452 | 0 |
| TAG56_6_FA20_5_uM | NA | 1.358 | 0 |
| TAG56_6_FA22_4_uM | NA | 1.771 | 0 |
| TAG56_6_FA22_5_uM | NA | 1.637 | 0 |
| TAG56_6_FA22_6_uM | NA | 1.579 | 0 |
| TAG56_7_FA16_0_uM | NA | 1.648 | 0 |
| TAG56_7_FA16_1_uM | NA | 1.62 | 0 |
| TAG56_7_FA18_0_uM | NA | 1.564 | 0 |
| TAG56_7_FA18_1_uM | NA | 1.513 | 0 |
| TAG56_7_FA18_2_uM | NA | 1.53 | 0 |
| TAG56_7_FA18_3_uM | NA | 1.47 | 0 |
| TAG56_7_FA20_3_uM | NA | 1.357 | 0 |
| TAG56_7_FA20_4_uM | NA | 1.381 | 0 |
| TAG56_7_FA20_5_uM | NA | 1.301 | 0 |
| TAG56_7_FA22_4_uM | NA | 1.725 | 0 |
| TAG56_7_FA22_5_uM | NA | 1.72 | 0 |
| TAG56_7_FA22_6_uM | NA | 1.559 | 0 |
| TAG56_8_FA16_1_uM | NA | 1.465 | 0 |
| TAG56_8_FA18_1_uM | NA | 1.432 | 0 |
| TAG56_8_FA18_2_uM | NA | 1.46 | 0 |
| TAG56_8_FA18_3_uM | NA | 1.376 | 0 |
| TAG56_8_FA20_4_uM | NA | 1.417 | 0 |
| TAG56_8_FA22_5_uM | NA | 1.619 | 0 |
| TAG56_8_FA22_6_uM | NA | 1.576 | 0 |
| TAG56_9_FA18_3_uM | NA | 1.273 | 0 |
| TAG56_9_FA22_6_uM | NA | 1.488 | 0 |
| TAG57_2_FA18_1_uM | NA | 1.334 | 0 |
| TAG57_8_FA22_6_uM | NA | 1.389 | 0 |
| TAG57_9_FA22_6_uM | NA | 1.517 | 0 |
| TAG58_10_FA22_6_uM | NA | 1.313 | 0 |
| TAG58_3_FA18_1_uM | -1.118E-05 | NA | 0 |
| TAG58_5_FA18_1_uM | NA | 1.558 | 0 |
| TAG58_6_FA16_0_uM | NA | 1.573 | 0 |
| TAG58_6_FA18_0_uM | NA | 1.46 | 0 |
| TAG58_6_FA18_1_uM | NA | 1.539 | 0 |
| TAG58_6_FA22_4_uM | NA | 1.5 | 0 |
| TAG58_6_FA22_5_uM | NA | 1.594 | 0 |
| TAG58_7_FA16_0_uM | NA | 1.698 | 0 |
| TAG58_7_FA18_0_uM | NA | 1.5 | 0 |
| TAG58_7_FA18_1_uM | NA | 1.543 | 0 |
| TAG58_7_FA18_2_uM | NA | 1.538 | 0 |
| TAG58_7_FA20_4_uM | NA | 1.305 | 0 |
| TAG58_7_FA22_4_uM | NA | 1.537 | 0 |
| TAG58_7_FA22_6_uM | NA | 1.477 | 0 |
| TAG58_8_FA18_1_uM | NA | 1.37 | 0 |
| TAG58_8_FA18_2_uM | NA | 1.477 | 0 |
| TAG58_8_FA20_4_uM | NA | 1.305 | 0 |
| TAG58_8_FA22_5_uM | NA | 1.434 | 0 |
| TAG58_8_FA22_6_uM | NA | 1.382 | 0 |
| TAG58_9_FA18_1_uM | NA | 1.317 | 0 |
| TAG58_9_FA18_2_uM | NA | 1.298 | 0 |
| TAG58_9_FA20_4_uM | 0.05853665 | NA | 0 |
| TAG58_9_FA22_5_uM | NA | 1.434 | 0 |
| TAG60_10_FA22_6_uM | -0.1105091 | NA | 0 |

^1^Molecular lipid species were log2 transformed and z-scaled

^2^Significant associations in individual regression are those with an FDR (BH) adjusted p-value < 0.05

^3^Interval censored Cox proportional hazards models with a Weibull distribution adjusted for sociodemographics, health behaviors, and clinical variables

^4^Penalized (LASSO) regression model trained on a case-balanced 0.7 data subset with 10 fold cross-validation

Supplemental Figure 7. Weighted LRS derived from penalized (LASSO) regression model of molecular lipid species. LASSO regression model of molecular lipid species on incident diabetes trained on 0.7 case-balanced data (n cases/non-cases = 423/448). LRS comprised of LASSO-selected molecular lipid species, weighted by the beta coefficients. Color (y-axis) indicates lipid class. X-axis is the beta coefficient (weight) of the lipid species in the LRS.

Supplemental Figure 8. AUC of the LRS derived from LASSO regression of molecular lipid species in the 0.7 training data (A) and the 0.3 testing data (B). Color indicates model specifications. Black = LRS, pink = health/clinical variables [physical activity score (continuous) + smoking (current yes/no) + diet (food groups, APDQS, total energy) + eGFR (continuous) + BMI (continuous) + hypertension (yes/no) + lipid-lowering medication use (yes/no)], green = sociodemographics [field center (Birmingham, AL; Chicago, IL; Minneapolis, MN; Oakland, CA) + age (continuous) + sex (male/female) + self-reported race (Black/White) + highest education attained (continuous years)], blue = sociodemographics + health/clinical variables, red = sociodemographics + health/clinical variables + LRS. The true positive rate (sensitivity) and false positive rate (1-specificity) is plotted on the y-axis and x-axis, respectively. AUCs for each model are noted in the figure legend in parentheses. Higher AUCs indicate increased ability of the model in discriminating 15-year incident diabetes cases from non-cases.


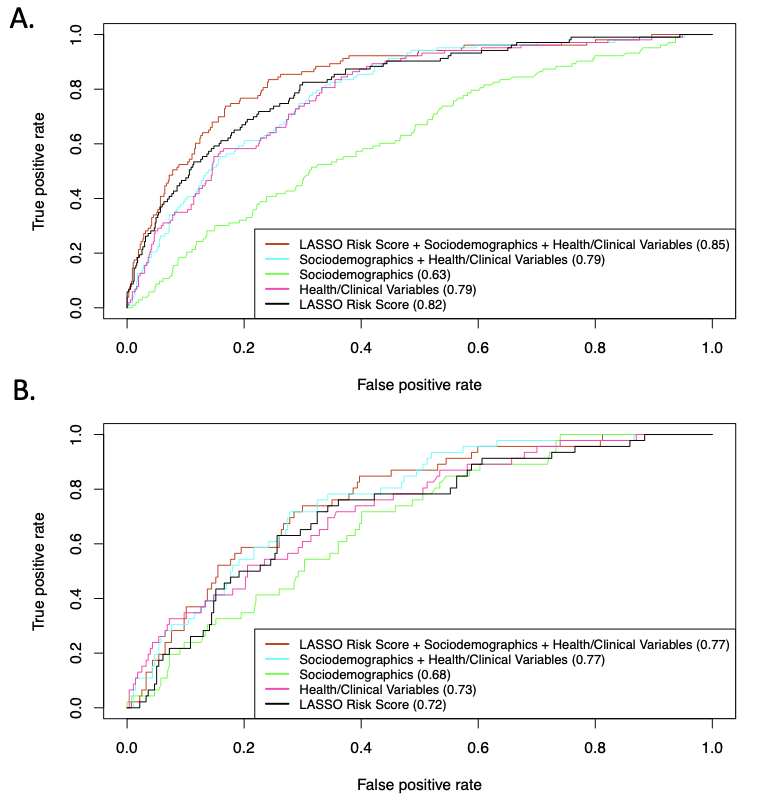

Supplement: Supplemental Material [file mmc1.docx]
