# Supplementary material for: Plasma lipidomics and 15-year risk of incident diabetes: a coronary artery risk development in young adults study
Source: J Lipid Res. 2026 Jan 30;67(3):100992. doi: 10.1016/j.jlr.2026.100992 (PMC12955097; doi:10.1016/j.jlr.2026.100992)
Supplement: Technical Supplement for Methods [file mmc2.docx]

Technical Supplement for Methods

STUDY SAMPLE

We used data from the U.S. population-based CARDIA study, which was established to study the evolution of cardiometabolic disease risk through adulthood. At baseline (1985-86), participants, aged 18-30 years, were recruited from four clinical field centers: Minneapolis, MN, Chicago, IL, Birmingham, AL, and Oakland, CA. Participants were recruited to achieve rough balance, within-field center, by age (18-24 and 25-30), sex (male/female), self-reported race (Black/White), and education (£high school graduate and >high school). This recruitment strategy ensured that there were not systemic regional (field center) differences with respect to age, sex, race, or education. There was a baseline sample of 5,115 participants (48% White, 45% male, 55% 25-30 years old, 60% > high school education). The majority of the baseline study sample provided informed consent and attended follow-up examinations (Y2 = 90%, Y5 = 86%, Y7 = 81%, Y10 = 79%, Y15 = 74%, Y20 = 72%, Y25 = 72%, Y30 = 71%, and Y35 = 67%). Details on CARDIA have been previously published. ^1^

LIPIDOMIC MEASURES

Lipidomic data were generated from stored plasma samples (-70°C) using the Metabolon Complex Lipids Targeted Panel. Samples were overnight shipped from the CARDIA repository to Metabolon on dry ice. Lipids were quantified using infusion-MS analysis with internal standards. This panel provided data on 1,125 lipid species across 14 lipid classes (ceramides, cholesteryl esters, diacylglycerols, dihydroceramides, hexosylceramides, lactosylceramides, lysophosphatidylcholines lysophosphatidylethanolamines, monoacylglycerols, phosphatidylcholines, phosphatidylethanolamines, phosphatidylinositols, sphingomyelins, and triacylglycerols). Details on the methods used to quantify lipid species have been previously published by Metabolon. ^2^

Throughout analysis and writing, we used standard lipidomic classifications and nomenclature. Adhering to standard lipidomic classifications and nomenclature ensures correct reporting of MS data, increases comparability to existing literature, and allows greater clarity in interpreting the data. We followed Lipid Metabolites and Pathways Strategy (LIPID MAPS^®^) standards. LIPID MAPS^®^ is an NIH-funded initiative for website and database development, to ensure comparability across studies. This free resource provides multiple resources related to lipidomic classifications and nomenclature. Details on LIPID MAPS^®^ and updated (2020) lipidomic data standards have been previously published. ^3^

COVARIATE MEASURES

CARDIA utilizes standardized and validated questionnaires to assess physical activity and dietary intake.

“Self-reported physical activity was measured using the validated CARDIA Physical Activity History questionnaire at each examination ([Gabriel et al., 2014](https://pmc.ncbi.nlm.nih.gov/articles/PMC8318903/#b0070), [Jacobs et al., 1993](https://pmc.ncbi.nlm.nih.gov/articles/PMC8318903/#b0100), [Jacobs et al., 1989](https://pmc.ncbi.nlm.nih.gov/articles/PMC8318903/#b0105)). The questionnaire asks about participation in 13 moderate physical activity (MPA, 5 out of the 13 activity types, including nonstrenuous sports, walking and hiking, golfing and bowling, home exercises or calisthenics, and home maintenance or gardening) and vigorous-intensity physical activity (VPA, 8 out of the 13 activity types, including running or jogging; racquet sports; biking; swimming; exercise or dance class; job lifting, carrying, or digging; shoveling or lifting during leisure; and strenuous sports) types over the previous year. Each activity type was assigned an intensity score (ranging from 3 to 8 metabolic equivalents) and a duration threshold (ranging from 2 to 5 h/week), above which participation was considered to be frequent. A score for each activity type was calculated using a computer algorithm based on the intensity of the activity, months of participation, and a weighting factor representing months with a minimum weekly duration. The total activity score was the sum of scores for all activities, expressed in exercise units, which represents the usual level of activity over the previous 12 months.”{Gabriel, 2014 #1849}

The physical activity history questionnaire captures participant participation in 13 moderate and vigorous activities over the past 3 months. Reported frequency and intensity for each activity are integrated for a measure of total physical activity. Further description and validation of the physical activity scores are published in Jacobs, et. al. ^4^ Dietary intake in CARDIA is assessed by an interviewer-administered dietary history questionnaire that captures usual intake of foods over the previous month. The diet history was based on a food frequency questionnaire, with additional queries related to food preparation. Compound foods, such as lasagna and soups, are decomposed into their component foods. The assessment provided data on over 1,600 food items across 100 food categories. The University of Minnesota Nutrition Coordinating Center assigned each food to one of 166 food groups, which were further collapsed into 46 food groups (e.g., regular red meat, lean red meat). Each food group was defined as either adverse (13), neutral (13), or beneficial (20) to health, based on an *a priori* evaluation of the literature and current dietary guidelines. Food groups were split into sample-based quintiles, and participants received a score based on their level of consumption (0-4, with ‘0’ indicating the lowest quintile of consumption, and ‘4’ indicating the highest). For foods with greater than 25% non-consumers, we assigned a ‘0’ to non-consumers and split consumers into quartiles (1-4). For calculation of the diet quality score, foods considered adversely related to health were reverse scored: lowest quintile of intake assigned a ‘4,’ highest level of intake assigned a ‘0.’ The *A Priori* Diet Quality Score (APDQS) was derived by summing food-specific scores across foods designated ‘adverse’ or ‘beneficial’ (‘neutral’ foods did not contribute to the APDQS). The APDQS had a theoretical maximum score of 132. Further details on CARDIAs diet history assessment, evaluation, and APDQS derivation have been previously published. ^5,6^

Estimated glomerular filtration rate (eGFR) was calculated using the Chronic Kidney Disease Epidemiology Collaboration (CKD-EPI) equation. In this equation, eGFR is calculated from serum creatinine, age, sex, race, and body weight. Details on the rationale and accuracy of this equation are discussed in Levey, et. al. ^7^

STATISTICAL ANALYSIS

In individual regression models of diabetes on lipid, we adjusted for multiple comparisons using the Benjamini-Hochberg approach to method the false discovery rate. ^8^

We conducted statistical analyses in R, utilizing the “ROSE” and “glmnet” packages for generating case-balanced data and for least absolute shrinkage and selection operator (LASSO) regression, respectively. Supervised modeling approaches that classify cases from non-cases have limited performance when the distribution of cases is unbalanced. To improve performance of such methods, case-balanced samples can be generated using a variety of statistical approaches. The “ROSE” R package generates a case-balanced sample using a smoothed bootstrap approach. ^9^ LASSO regression is a penalized model that selects coefficients of correlated predictors and shrinks the others to zero. We conducted LASSO regression using the “glmnet” R package. ^10^

References

1. Friedman GD, Cutter GR, Donahue RP, et al. CARDIA: study design, recruitment, and some characteristics of the examined subjects. *J Clin Epidemiol.* 1988;41(11):1105-1116.

2. Complex Lipids Targeted Panel. In. Metabolon; 2021:3. https://www.metabolon.com/wp-content/uploads/2023/03/Metabolon-Panel-Complex-Lipids.pdf

3. Liebisch G, Fahy E, Aoki J, et al. Update on LIPID MAPS classification, nomenclature, and shorthand notation for MS-derived lipid structures. *Journal of Lipid Research.* 2020;61(12):1539-1555.

4. Jacobs DR, Jr., Hahn LP, Haskell WL, Pirie P, Sidney S. Validity and Reliability of Short Physical Activity History: Cardia and the Minnesota Heart Health Program. *J Cardiopulm Rehabil.* 1989;9(11):448-459.

5. McDonald A, Van Horn LV, Slattery ML, et al. The CARDIA dietary history: development, implementation, and evaluation. *Journal of the American Dietetic Association.* 1991;91 9:1104-1112.

6. Sijtsma FP, Meyer KA, Steffen LM, et al. Longitudinal trends in diet and effects of sex, race, and education on dietary quality score change: the Coronary Artery Risk Development in Young Adults study. *The American journal of clinical nutrition.* 2012;95(3):580-586.

7. Levey AS, Stevens LA. Estimating GFR using the CKD Epidemiology Collaboration (CKD-EPI) creatinine equation: more accurate GFR estimates, lower CKD prevalence estimates, and better risk predictions. *Am J Kidney Dis.* 2010;55(4):622-627.

8. Benjamini Y, Hochberg Y. Controlling the false discovery rate: a practical and powerful approach to multiple testing. *Journal of the Royal statistical society: series B (Methodological).* 1995;57(1):289-300.

9. Lunardon N, Menardi G, Torelli N. ROSE: a package for binary imbalanced learning. 2014.

10. Zou H, Hastie T. Regularization and variable selection via the elastic net. *Journal of the Royal Statistical Society Series B: Statistical Methodology.* 2005;67(2):301-320.
